# Limiting glutamine utilization activates a GCN2/TRAIL-R2/Caspase-8 apoptotic pathway in glutamine-addicted tumor cells

**DOI:** 10.1038/s41419-022-05346-y

**Published:** 2022-10-27

**Authors:** Rosario Yerbes, Rocío Mora-Molina, F. Javier Fernández-Farrán, Laura Hiraldo, Abelardo López-Rivas, Carmen Palacios

**Affiliations:** 1 Centro Andaluz de Biología Molecular y Medicina Regenerativa-CABIMER, CSIC-Universidad de Sevilla-Universidad Pablo de Olavide, Avda Américo Vespucio 24, 41092 Sevilla, Spain; 2 Centro de Investigación Biomédica en Red-Oncología (CIBERONC), Carlos III Health Institute, Madrid, Spain

**Keywords:** Cancer metabolism, Apoptosis

## Abstract

Oncogenic transformation leads to changes in glutamine metabolism that make transformed cells highly dependent on glutamine for anabolic growth and survival. Herein, we investigated the cell death mechanism activated in glutamine-addicted tumor cells in response to the limitation of glutamine metabolism. We show that glutamine starvation triggers a FADD and caspase-8-dependent and mitochondria-operated apoptotic program in tumor cells that involves the pro-apoptotic TNF-related apoptosis-inducing ligand receptor 2 (TRAIL-R2), but is independent of its cognate ligand TRAIL. In glutamine-depleted tumor cells, activation of the amino acid-sensing general control nonderepressible-2 kinase (GCN2) is responsible for TRAIL-R2 upregulation, caspase-8 activation, and apoptotic cell death. Interestingly, GCN2-dependent ISR signaling induced by methionine starvation also leads to TRAIL-R2 upregulation and apoptosis. Moreover, pharmacological inhibition of transaminases activates a GCN2 and TRAIL-R2-dependent apoptotic mechanism that is inhibited by non-essential amino acids (NEAA). In addition, metabolic stress upon glutamine deprivation also results in GCN2-independent FLICE-inhibitory protein (FLIP) downregulation facilitating caspase-8 activation and apoptosis. Importantly, downregulation of the long FLIP splice form (FLIP_L_) and apoptosis upon glutamine deprivation are inhibited in the presence of a membrane-permeable α-ketoglutarate. Collectively, our data support a model in which limiting glutamine utilization in glutamine-addicted tumor cells triggers a previously unknown cell death mechanism regulated by GCN2 that involves the TRAIL-R2-mediated activation of the extrinsic apoptotic pathway.

## Introduction

The so-called extrinsic pathway of apoptosis utilizes membrane-localized death receptors of the tumor necrosis factor (TNF) receptor superfamily to activate the caspases cascade and apoptosis upon ligand binding [1]. Tumor necrosis factor-related apoptosis-inducing ligand (TRAIL) is a member of the TNF family [2] that induces apoptosis selectively in a wide variety of cancer cells upon binding to pro-apoptotic receptors [3, 4]. Upon TRAIL receptors activation, there is the formation of a death-inducing signaling complex (DISC), which is required for caspase-8 activation and that includes the receptor itself, the adapter molecule FADD and procaspase-8 [5]. The short-lived protein cFLIP, homologue of vFLIP in vertebrate cells [6], inhibits caspase-8 processing and activation being their levels critical controllers of DISC output [7]. Interestingly, in recent years there have been some reports on the involvement of TRAIL receptors in cell death induced in response to stress in tumor cells [8–12].

The increased uptake and metabolism of glutamine in tumor cells provide these cells with intermediates for nucleotide and protein synthesis, redox homeostasis, and mitochondrial energy metabolism required for proliferation [13]. Increased consumption of glutamine by proliferating tumor cells, together with an abnormal vasculature, leads to selective loss of glutamine in the tumor microenvironment [14]. Moreover, a recent study using metabolomics analysis has shown that comparing tumor patient samples with benign adjacent tissue specimens, glutamine is one of the most strongly depleted metabolites in tumors [15]. Furthermore, the core region of solid tumors displayed glutamine deficiency when compared with other amino acids [16].

In response to amino acid starvation cells can activate the integrated stress response (ISR) aiming at resolving stress and reestablishing homeostasis to maintain cell viability and proliferation [17]. The initial event in this signaling response is the phosphorylation of eukaryotic translation initiation factor 2 alpha (eIF2α) by the GCN2 kinase to inhibit cap-dependent global protein synthesis while allowing the translation of specific mRNAs containing a short upstream open reading frame (uORF) in their 5’ untranslated region [18]. Activation of GCN2 lies at the center of the ISR, dictating how cells respond to detrimental conditions, such as those found in solid tumors. In this sense, GCN2 activation has a primary adaptive function being critical for tumor cell survival and proliferation in response to stress upon nutrient deprivation [19–21]. It is also clear that under prolonged starvation, activation of the ISR can lead to apoptosis to eliminate chronically stressed cells [22–25] although the underlying signaling mechanism remains hitherto poorly elucidated.

Here, we have examined the role of GCN2 in cell fate decisions after either glutamine starvation or pharmacological inhibition of transaminases in glutamine-addicted tumor cell lines. We demonstrate that restricting glutamine utilization in tumor cells induces a GCN2-dependent signaling pathway leading to TRAIL-R2 upregulation that together with GCN2-independent FLIP downregulation results in caspase-8 activation and apoptotic cell death.

## Results

### Glutamine deprivation induces tumor cell death through a mitochondria-operated pathway of apoptosis

**Fig. 1:**
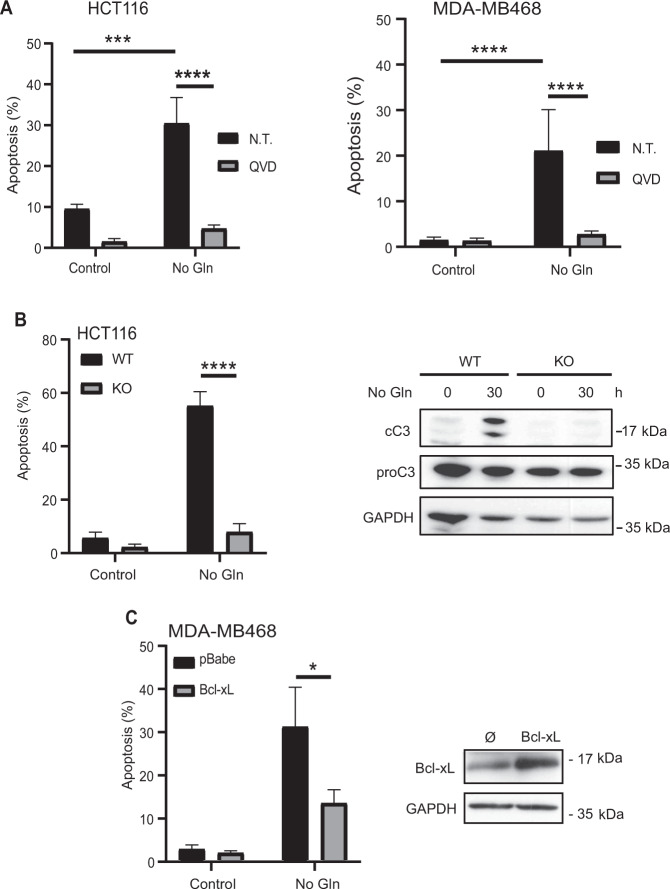
Glutamine deprivation induces apoptosis in tumor cell lines of different origins by a mitochondria-operated pathway. **A** HCT116 (left) or MDA-MB468 (right) cells were cultured for 48 h in medium with or without glutamine (Gln) in the presence or absence of Q-VD-OPh (20 µmol/L). Apoptosis was determined as described in Materials and Methods section. **B** Apoptosis was assessed in HCT116 WT or Bax/Bak KO cells cultured in the presence or absence of glutamine for 48 h (left panel). Procaspase-3 (proC3) and cleaved caspase-3 (cC3) levels were assessed by Western blotting in HCT116 WT or Bax/Bak KO cells starved of glutamine for 30 h (right panel). GAPDH was used as protein-loading control. **C** pBabe or Bcl-xL-overexpressing MDA-MB468 cells were cultured for 48 h in medium with or without glutamine and apoptosis assessed as described in **A**. Bcl-xL overexpression was confirmed by Western blotting. Data are presented as mean ± SD from at least three independent experiments. *P < 0.05; ***P < 0.001; ****P < 0.0001; two-way ANOVA test. Tukey’s multiple comparison test.

Tumor cells heavily rely on extracellular glutamine to support cell survival and growth [13]. However, tumor microenvironment is often subject to fluctuations in glutamine levels as tumor growth exceeds the delivery capabilities of the existing vasculature, resulting in metabolic stress [14]. Prior to tumor adaptation to survive metabolic stress, glutamine limitation may lead to tumor cell death [16, 26, 27], although the underlying mechanism remains largely unknown. We have studied the cell death response to glutamine starvation by determining the induction of apoptosis in glutamine addicted-tumor cell lines growing in glutamine-free medium. Glutamine starvation induced a caspase-dependent apoptotic program in colorectal carcinoma (HCT116) and triple-negative breast carcinoma (MDA-MB468) cell lines (Fig. 1A). Next, we investigated the role of the mitochondria in the induction of apoptosis upon glutamine shortage. To this end, HCT116 Bax/Bak KO cells were subject to glutamine deprivation and apoptosis was assessed. Results shown in Fig. 1B illustrates that in Bax/Bak KO cells apoptosis was completely blocked as determined by the analysis of hypodiploid cells (left panel) and caspase-3 activation (right panel). Likewise, MDA-MB468 breast tumor cells over-expressing the anti-apoptotic Bcl-xL protein were also markedly resistant to glutamine deprivation (Fig. 1C).

### GCN2 plays a pro-apoptotic role in cell death induced by glutamine deprivation

**Fig. 2:**
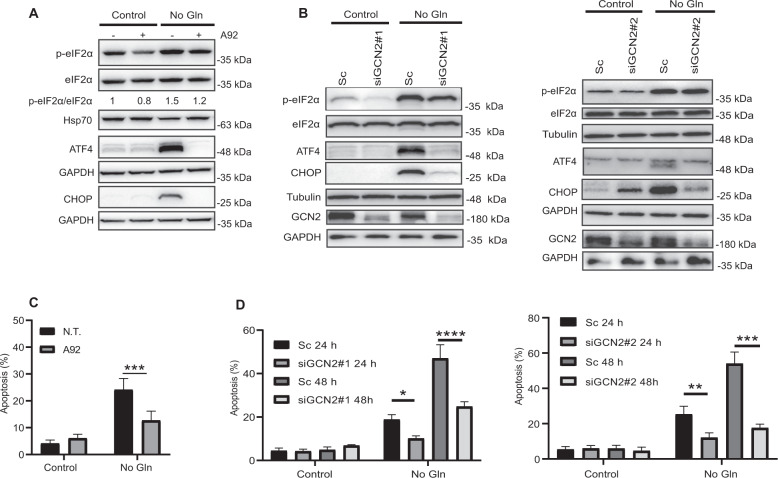
GCN2 is playing a pro-apoptotic role in cell death induced by glutamine deprivation. **A** HCT116 cells were cultured for 15 h in the presence or absence of glutamine, with or without 1 µM A92. Western blotting was performed to examine eIF2-α phosphorylation, eIF2-α, ATF4, and CHOP levels. Levels of phosphorylated eIF2-α relative to total eIF2-α protein were quantified by ImageQuant TL analysis software (Cytiva, USA). GAPDH was used as protein-loading control. **B** HCT116 cells were transfected for 48 h with either a Scrambled oligonucleotide (Sc) or two different siRNAs targeting GCN2 (siGCN2#1 and #2). After transfection, cells were incubated for 24 h in complete or glutamine-depleted medium and levels of eIF2α phosphorylation, eIF2α, GCN2, ATF4 and CHOP were assessed by Western blotting. **C** HCT116 cells were incubated for 24 h in medium with or without glutamine in the presence or absence of 1 µM A92 and apoptosis assessed as described in “Materials and methods” section. **D** HCT116 cells were transfected for 48 h as described in **B**. After transfection, cells were incubated for 24 or 48 h in complete or glutamine-depleted medium and apoptosis was then assessed. In **C** and **D**, data are presented as mean ± SD from at least three independent experiments. *P < 0.05; **P < 0.01; ***P < 0.001; ****P < 0.0001; two-way ANOVA test. Tukey’s multiple comparison test.

In response to different environmental and pathological stress conditions eukaryotic cells activate an adaptive pathway, called the integrated stress response (ISR), to restore cellular homeostasis [28, 29]. The core event in this pathway is the phosphorylation of eukaryotic translation initiation factor 2 alpha (eIF2α), which leads to inhibition of general cap-dependent protein translation, but induces cap-independent translation of selected mRNAs, such as the transcription factor ATF4, that promote cellular recovery. However, under chronic or severe stress, cell death can be induced by transcriptional activation of ATF4 target gene CHOP (GADD153) [30]. Under conditions of lack of amino acids in the extracellular medium a stress response is induced involving eIF2α phosphorylation by the general control non-depressible 2 (GCN2) kinase, which gets activated by uncharged tRNAs [31] although tRNA-independent mechanisms may be also involved [32, 33]. To find out whether GCN2-eIF2α-ATF4-CHOP pathway was activated in HCT116 tumor cells deprived of glutamine, cells were incubated in glutamine-free medium in the presence or absence of the GCN2 inhibitor A92 [20]. As shown in Fig. 2A, glutamine starvation in HCT116 cells induced eIF2α phosphorylation as well as upregulation of the transcription factors ATF4 and CHOP. All these events were reduced or inhibited in the presence of A92, suggesting the involvement of GCN2 in the activation of the ISR by glutamine limitation in HCT116 cells.

To further demonstrate the role of GCN2 in the stress response activated by glutamine deprivation, we genetically depleted GCN2 by RNA interference with two different siRNA oligonucleotides. GCN2 knockdown attenuated eIF2α phosphorylation and prevented ATF4 and CHOP induction in cells deprived of glutamine (Fig. 2B, left and right panels). Importantly, either inhibiting or silencing GCN2 led to significant inhibition of apoptosis in glutamine-deprived HCT116 tumor cells (Fig. 2C, D).

### GCN2-mediated TRAIL-R2 upregulation and activation of the extrinsic apoptotic pathway upon glutamine deprivation in tumor cells

**Fig. 3:**
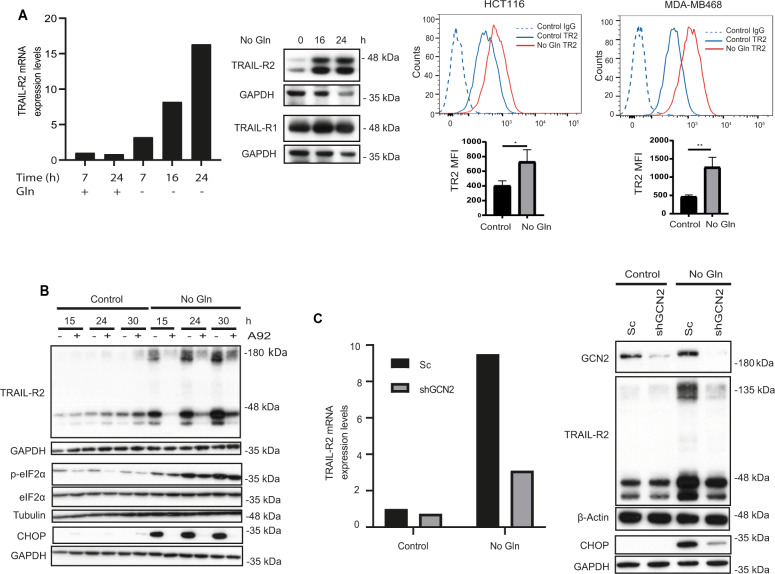
GCN2-dependent TRAIL-R2 upregulation upon glutamine deprivation in tumor cells. **A** HCT116 cells were cultured in the presence or absence of glutamine for the indicated times and TRAIL-R2 mRNA levels were measured by RT-qPCR (left panel). TRAIL-R2 and TRAIL-R1 protein levels were assessed by Western blotting (middle panel). GAPDH was used as protein-loading control. Cell surface TRAIL-R2 levels (right panel) were also analyzed in HCT116 and MDA-MB468 cell lines after 24 h of glutamine deprivation in the presence of 20 µM Q-VD-OPh, as described in Materials and Methods (MFI: geometric mean fluorescent intensity). **B** HCT116 cells were incubated for the indicated times in medium with or without glutamine, in the presence or absence of 1 µM A92. Western blotting was performed to determine TRAIL-R2, p-eIF2α, eIF2α and CHOP levels. **C** HCT116 cells stably expressing either a scrambled (Sc) or a GCN2 targeting sequence (shGCN2), were incubated for 17 h in the presence or absence of glutamine. Following this incubation, TRAIL-R2 mRNA levels were measured by RT-qPCR (left panel) and GCN2, CHOP and TRAIL-R2 protein levels were assessed by Western blotting (right panel).

Expression of pro-apoptotic receptor TRAIL-R2 is up-regulated in cells undergoing different forms of stress through a signaling pathway involving CHOP, a transcription factor for TRAIL-R2 gene expression [8–10, 34, 35]. To elucidate the mechanism underlying the pro-apoptotic role of GCN2 activation in glutamine-starved tumor cells, we initially determined the expression of pro-apoptotic TRAIL-R2 receptor upon glutamine deprivation. As shown in Fig. 3A, incubation of HCT116 cells in glutamine-free medium markedly up-regulated TRAIL-R2/DR5 mRNA and protein levels. Increased cell surface expression of this receptor upon glutamine limitation was also observed in HCT116 and MDA-MB468 cells (Fig. 3A, right panel). In contrast, TRAIL-R1 protein levels remained unchanged upon glutamine limitation (Fig. 3A, middle panel). Interestingly, induction of TRAIL-R2 following glutamine deprivation was abolished in the presence of the GCN2 inhibitor A92 (Fig. 3B). To further validate the role of GCN2 in controlling TRAIL-R2 levels under metabolic stress, we determined TRAIL-R2 expression upon glutamine deprivation in HCT116 cells in which GCN2 expression had been stably knockdown by shGCN2. Results shown in Fig. 3C demonstrate that mRNA and protein levels of TRAIL-R2 were markedly reduced in shGCN2 cells subject to glutamine starvation, confirming that TRAIL-R2 levels are controlled by GCN2 when these tumor cells are deprived of glutamine.

Involvement of GCN2 in TRAIL-R2 upregulation and apoptosis upon glutamine limitation was also investigated in the triple-negative breast tumor cell line MDA-MB468. Interestingly, glutamine deprivation-induced cell death was significantly inhibited by GCN2 knockdown (Fig. S1A, left panel). Furthermore, GCN2 knockdown by siRNA markedly inhibited the ISR and TRAIL-R2 upregulation in glutamine-deprived MDA-MB468 cells (Fig. S1A, right panel).

Taken together, these results clearly indicate that GCN2 is playing a pro-apoptotic role in cell death induced by glutamine deprivation in these glutamine-addicted colon carcinoma and breast tumor cell lines.

**Fig. 4:**
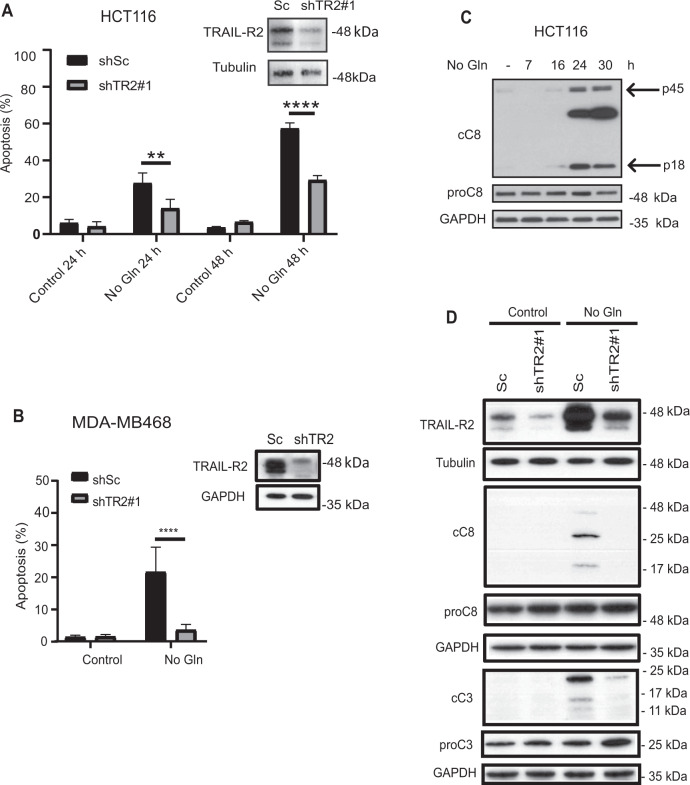
Role of TRAIL-R2 in apoptosis induced by glutamine deprivation in tumor cells. **A** (HCT116) or **B** (MDA-MB468) cells stably expressing a scrambled (shSc) or a TRAIL-R2 targeting shRNA (shTRAIL-R2#1) were cultured with or without glutamine and apoptosis was assessed at the indicated times (HCT116) or 48 h (MDA-MB468). TRAIL-R2 knockdown was determined by Western blotting. Tubulin and GAPDH were used as protein-loading controls. Data are presented as mean ± SD from at least three independent experiments. **P < 0.01; ****P < 0.0001; two-way ANOVA test. Tukey’s multiple comparison test. **C** Procaspase-8 levels and caspase-8 activation (cC8) in HCT116 cells incubated in the presence or absence of glutamine for the indicated times. **D** Scrambled (Sc) or shTRAIL-R2#1 HCT116 cells were cultured in the presence or absence of glutamine for 24 h. Following this incubation, TRAIL-R2 levels, caspase-8 and caspase-3 activation, as well as procaspase-8 or procaspase-3 levels were assessed by Western blotting.

We next sought to determine the role of GCN2-mediated TRAIL-R2 upregulation in glutamine deprivation-induced apoptosis. Strikingly, HCT116 cells in which TRAIL-R2 expression was silenced by shRNA showed a marked resistance to apoptosis induced by glutamine deprivation (Figs. 4A and S1B). This role of TRAIL-R2 in apoptosis induced by glutamine starvation was also confirmed in MDA-MB468 cells (Fig. 4B). To examine the role of TRAIL in TRAIL-R2-mediated apoptosis upon glutamine deprivation, we initially used a soluble TRAIL-R2-Fc fusion protein that completely blocks extracellular TRAIL-induced apoptosis (Fig. S1C, left panel). However, data shown in figure S1C (right panel) indicate that cell death induced by glutamine starvation is independent of exogenous TRAIL in HCT116 cells. Due to undetectable levels of this ligand in control and glutamine-deprived HCT116 cells, we could not completely rule out that endogenous TRAIL may play a role in apoptosis induced by glutamine limitation in these tumor cells. However, silencing endogenous TRAIL expression in MDA-MB468 cells further demonstrates that apoptosis induced by glutamine removal is independent of this ligand (Fig. S1D). Initiator caspase-8 is a key component of the extrinsic apoptosis signaling induced upon death receptors activation by their cognate ligands [1], Ligand-independent activation of caspase-8 has also been reported in cells exposed to a different type of environmental or endogenous stresses [9–12], including nutrient imbalance [36]. Interestingly, glutamine deprivation induced caspase-8 activation in HCT116 cells (Fig. 4C). Furthermore, activation of both caspase-8 and caspase-3 in response to glutamine starvation was abolished in shTRAIL-R2 HCT116 cells (Fig. 4D). Collectively, these data suggest that activation of TRAIL-R2 receptor in the absence of its cognate ligand TRAIL is a key event in apoptotic cell death induced upon metabolic stress by glutamine deprivation.

**Fig. 5:**
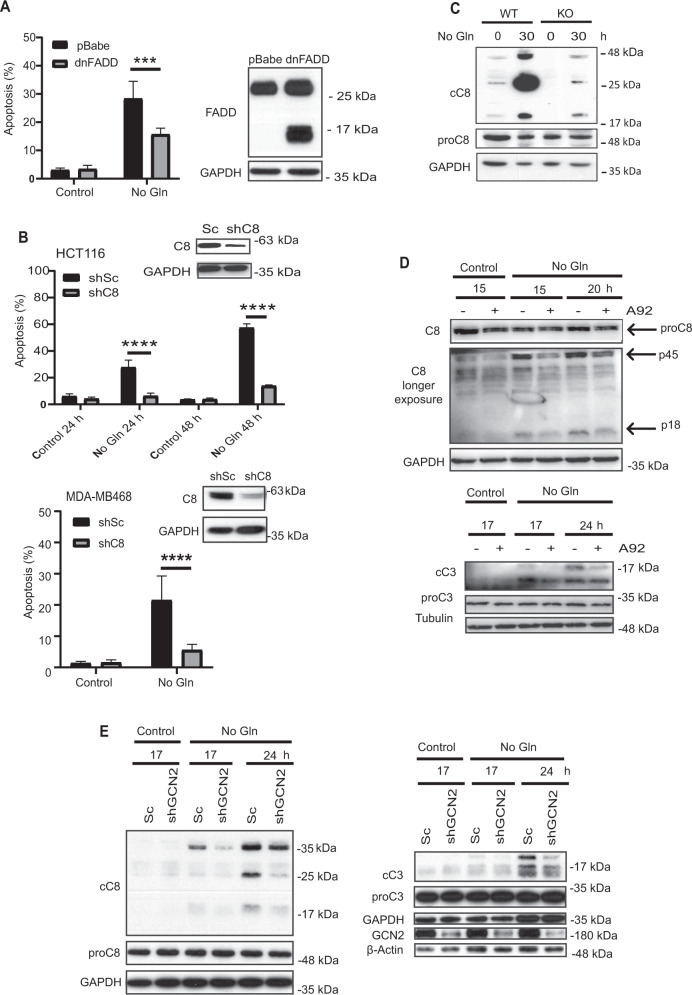
GCN2-mediated activation of the extrinsic apoptotic pathway upon glutamine deprivation in tumor cells. **A** Apoptosis was assessed in pBabe or dnFADD HCT116 cells cultured in the presence or absence of glutamine for 30 h. Endogenous FADD and dnFADD expression were assessed by Western-blotting. GAPDH was used as protein-loading control. **B** HCT116 (upper panel) or MDA-MB468 (lower panel) cells stably expressing a Scrambled or a caspase-8 targeting shRNA, were incubated in the presence or absence of glutamine and apoptosis was measured at the indicated times (HCT116) or 48 h (MDA-MB468). **C** HCT116 WT or Bax/Bak KO cells were cultured in the presence or absence of glutamine for 30 h. Following these treatments, procaspase-8 (proC8) and cleaved caspase-8 (cC8) were assessed by Western blotting. Data are presented as mean ± SD from at least three independent experiments. ***P < 0.001; ****P < 0. 0001; two-way ANOVA test. Tukey’s multiple comparison test. **D** HCT116 cells were incubated in medium with or without glutamine for the indicated times, in the presence or absence of 1 µM A92. Caspase-8 (upper panel) and caspase-3 (cC3) (lower panel) activation was assessed by Western blotting. **E** HCT116 cells stably expressing a scrambled oligonucleotide (shSc) or a GCN2 targeting shRNA (shGCN2) were cultured in the presence or absence of glutamine for the indicated times. Following these incubations, caspase-8 (cC8, left panel) and caspase-3 (cC3, right panel) activation, and GCN2 levels (right panel) were assessed by Western blotting.

Involvement of the extrinsic apoptotic pathway in apoptosis induced by glutamine deprivation was further verified in HCT116 cells overexpressing a dominant-inhibitory form of the adaptor protein FADD (dnFADD) [37], to disrupt the association between death receptors and caspase-8, thus impeding downstream apoptotic signaling. Cells over-expressing dnFADD were significantly more resistant than their corresponding controls to apoptosis induced upon glutamine deprivation (Fig. 5A). The contribution of the extrinsic pathway of apoptosis to cell death induced by glutamine deprivation was also assessed by stable knockdown of caspase-8 expression in HCT116 and MDA-MB468 cell lines with shRNA. As shown in Fig. 5B, silencing of caspase-8 expression markedly reduced the sensitivity to glutamine starvation in both tumor cell lines. These results were validated in HCT116 cells by silencing caspase-8 expression using a siRNA with a different sequence than the one used to generate shC8 cells (Fig. S1E). Thus, apoptosis induced in glutamine-starved cell lines depends on TRAIL-R2, FADD and Caspase-8, pointing to a role of the extrinsic pathway of apoptosis in this cell death process.

There is a growing body of evidence indicating that feedback amplification of apical apoptotic caspases by effector caspases is required for efficient apoptosis [38]. Thus, we next assessed the caspase-8 activation state in HCT116 Bax/Bak KO cells in which caspase-3 activation was inhibited following metabolic stress by glutamine deprivation. Importantly, there was a marked inhibition of caspase-8 activation in KO cells cultured in the absence of glutamine (Fig. 5C). However, caspase-8 processing was still observed in Bax/Bak KO cells deprived of glutamine, indicating that caspase-8 may get activated initially by TRAIL-R2 stimulation, although signal amplification through the mitochondrial pathway is required to complete the apoptotic process. Finally, caspase-8 activation in glutamine-deprived tumor cells was significantly inhibited in the presence of the GCN2 inhibitor, A92 (Fig. 5D, upper panel). GCN2 inhibition also reduced effector caspase-3 activation under the same conditions of metabolic stress (Fig. 5D, lower panel). GCN2 dependence of both caspase-8 and caspase-3 activation in cells deprived of glutamine was also demonstrated in HCT116 shGCN2 cells (Fig. 5E).

Collectively, the above results demonstrated that in glutamine-addicted tumor cells depletion of glutamine triggers an apoptotic response mediated by the GCN2/TRAIL-R2/Caspase-8 signaling pathway. To ascertain whether sustained activation of the ISR by amino acid deprivation is sufficient to elicit an apoptotic response involving this pathway, we examined whether methionine deprivation in glutamine-replete medium may also activate a GCN2-dependent TRAIL-R2 increase and apoptosis in HCT-116 cells. Data shown in Fig. S2A demonstrate that methionine starvation activates GCN2-dependent ISR signaling and TRAIL-R2 upregulation. Remarkably, silencing experiments clearly indicate that apoptosis induced upon methionine starvation also involves activation of a GCN2/TRAIL-R2/Caspase-8 pathway (Fig. S2B). Together, these data reveal that sustained amino acid starvation in these tumor cells triggers apoptosis through a previously unknown mechanism regulated by GCN2 that involves the TRAIL-R2-mediated activation of the extrinsic apoptotic pathway.

### Role of FLIP in cell death induced by glutamine deprivation

**Fig. 6:**
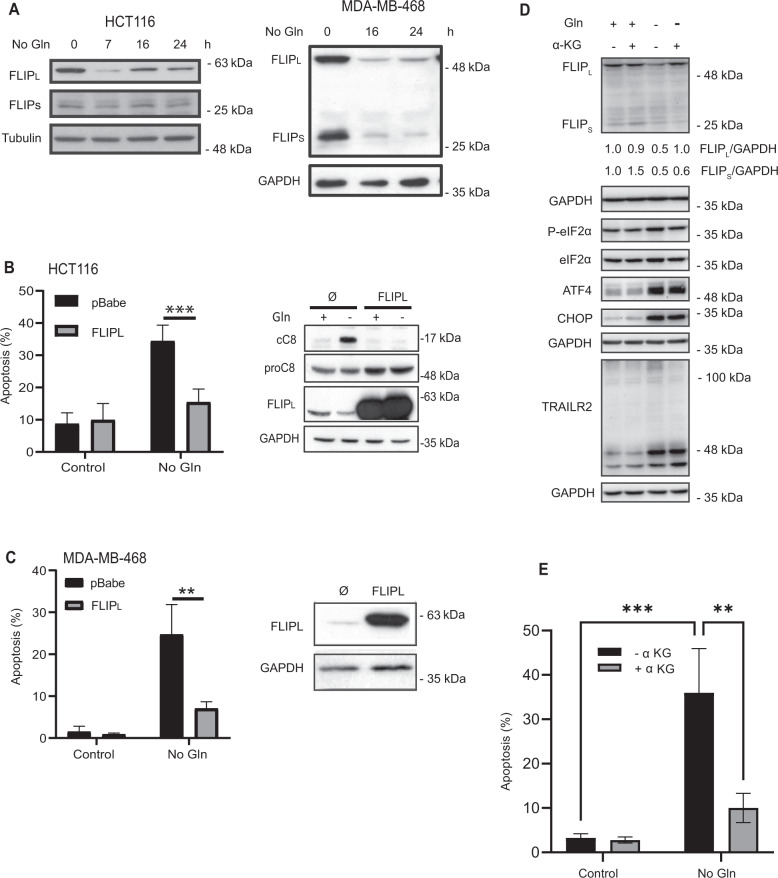
Role of FLIP in cell death induced by glutamine deprivation. **A** HCT116 (left panel) or MDA-MB468 (right panel) cells were cultured in the presence or absence of glutamine for the indicated times. Tubulin and GAPDH were used as protein-loading controls. Following these treatments, FLIP_L_ and FLIP_S_ levels were assessed by Western blotting. **B** Apoptosis was assessed in pBabe or FLIP_L_ overexpressing HCT116 cells cultured in the presence or absence of glutamine for 48 h (left panel). Procaspase-8 levels, caspase-8 activation (cC8) and FLIP_L_ overexpression were determined by Western-blotting after 30 h of glutamine starvation (right panel). **C** Apoptosis was assessed in pBabe or FLIPL-overexpressing MDA-MB468 cells cultured in the presence or absence of glutamine for 48 h. FLIP_L_ overexpression was detected by Western blotting. In **B** and **C** data are presented as mean ± SD from at least three independent experiments. **P < 0.01; ***P < 0.001; two-way ANOVA test. Tukey’s multiple comparison test. **D** HCT116 cells were cultured in the presence or absence of glutamine for 16 h, with or without dimethyl α-ketoglutarate (5 mM). FLIP levels, ISR activation and TRAIL-R2 upregulation were assessed by Western blotting. In **E** apoptosis was assessed in HCT116 cells cultured in the presence or absence of glutamine for 48 hours, with or without dimethyl α-ketoglutarate. Data are presented as mean ± SD from at least three independent experiments. **P < 0.01; ***P < 0.001; two-way ANOVA test. Tukey’s multiple comparison test.

The anti-apoptotic proteins FLIP_L_ and FLIP_S_ are key regulators of caspase-8 activation upon TRAIL receptors clustering by TRAIL [6, 39, 40]. To get further insight into the mechanism underlying glutamine deprivation-induced apoptosis, we first analyzed FLIP levels in cells starved from glutamine. As shown in Fig. 6A, FLIP_L_ expression was markedly down-regulated in both HCT116 and MDA-MB468 cells upon glutamine removal from the culture medium. Regarding FLIPs isoform, there were clear differences between HCT116 and MDA-MB468 cells in their response to glutamine deprivation, with a strong downregulation in MDA-MB468 cells and less pronounced effects in HCT116 cells. To assess whether the decrease in FLIP levels could be involved in cell death induced by glutamine deprivation, we generated bulk populations of HCT116 (Fig. 6B) or MDA-MB468 (Fig. 6C) cells overexpressing FLIP_L_ by infection with a FLIP_L_-encoding retroviral vector. Importantly, induction of apoptosis upon glutamine starvation was markedly reduced in both cell lines overexpressing FLIP_L_, confirming the role of the extrinsic apoptotic pathway in this cell death process. Moreover, in HCT116 cells overexpressing FLIP_L_ caspase-8 activation induced by glutamine depletion was completely inhibited (Fig. 6B, right panel). Together, these results suggest that downregulation of FLIP levels upon glutamine deprivation is also contributing to cell death induced by the GCN2-activated extrinsic apoptotic pathway in these tumor cells. Next, we determined whether GCN2 activation plays any role in the observed downregulation of FLIP levels in HCT116 cells subject to glutamine limitation. As shown in figure S3, FLIP loss upon glutamine deprivation was not prevented in cells lacking GCN2 (Fig. S3A, left panel) or treated with A92 (Fig. S3A, right panel). GCN2 knockdown or inhibition of GCN2 by A92 not only did not prevent FLIP_L_ downregulation upon glutamine deprivation but it caused a further decrease in FLIP levels, suggesting that a GCN2-independent mechanism is responsible for the loss of FLIP in glutamine-starved HCT116 cells.

Conversion of glutamine to α-ketoglutarate is a major metabolic fate of glutamine in proliferating normal and tumor cells [13]. To get further insight into the mechanism responsible for FLIP_L_ downregulation in HCT116 cells deprived of glutamine we investigated whether addition of permeable αKG (dmαKG) may prevent FLIP_L_ loss and cell death by apoptosis in glutamine-starved tumor cells. Interestingly, addition of dmαKG to cultures of glutamine-starved HCT116 cells markedly inhibited FLIP_L_ downregulation (Fig. 6D). In contrast, ISR activation and TRAIL-R2 upregulation were not affected by dmαKG (Fig. 6D). Importantly, data shown in Fig. 6E reveal that apoptosis upon glutamine deprivation was inhibited in the presence of dmαKG. Given the importance of maintaining FLIP_L_ levels to prevent apoptosis in cells deprived of glutamine (Fig. 6B, C), all these results suggest that the limitation of glutamine in glutamine-addicted tumor cells will lead, on the one hand, to the elevation of TRAIL-R2 levels mediated by the activation of the GCN2 pathway and, on the other hand, to the decrease of FLIP_L_ as a result of the metabolic defect caused by the loss of αKG. In this regard, although ectopic over-expression of TRAIL-R2 may induce apoptosis in tumor cells growing in glutamine-containing medium (Fig. S3B) silencing FLIP_L_ expression prior to ectopic TRAIL-R2 over-expression further increases caspase-8 activation and cell death by apoptosis under glutamine replete conditions (Fig. S3C, D). Altogether, our results support a model in which both TRAIL-R2 upregulation and FLIP downregulation are important events in glutamine deprivation-induced apoptosis in glutamine-addicted tumor cells.

### Pharmacological inhibition of glutamine-utilizing transaminases induces apoptosis depending on GCN2 and TRAIL-R2-mediated activation of the extrinsic apoptotic pathway

**Fig. 7:**
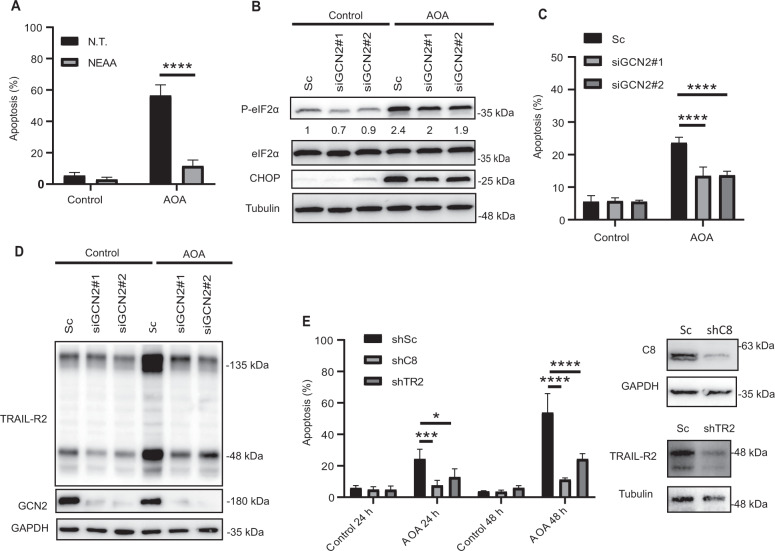
GCN2-dependent activation of the extrinsic pathway of apoptosis in tumor cells treated with AOA. **A** Apoptosis in HCT116 cells cultured for 48 h in complete medium with or without AOA in the presence or absence of non-essential amino acids (NEAA). **B**, **C** HCT116 cells were transfected either with a scrambled oligonucleotide (Sc) or with two different siRNAs targeting GCN2 (siGCN2#1 or #2). 48 hours after transfection, cells were either incubated for 16 h in the presence of absence of AOA to assess phosphorylated eIF2α, eIF2α and CHOP levels by Western blotting (**B**), or during 24 h to measure apoptosis (**C**)**. D** HCT116 cells were transfected and treated as described in **B**. TRAIL-R2 and GCN2 levels were assessed by Western blotting. GAPDH was used as protein-loading control. **E** HCT116 cells stably expressing scrambled, caspase-8, or TRAIL-R2 targeting shRNA, were cultured in the presence or absence of AOA, and apoptosis was measured at 24 or 48 h. Caspase 8 and TRAIL-R2 knockdown were determined by Western blotting. In **A**, **C** and **E**, data are presented as mean ± SD from at least three independent experiments. *P < 0.05; ***P < 0.001; ****P < 0.0001; two-way ANOVA test. Tukey’s multiple comparison test.

Proliferating cells rely on glutaminase (GLS) to produce glutamate, a critical product of glutamine catabolism which is used for driving the biosynthesis of NEAA, through the activity of transaminases. In this respect, highly proliferative human breast tumors display high transaminase expression, coupling glutamine consumption to NEAA synthesis to boost protein biosynthesis required for tumor cells survival and growth [41]. Moreover, glutamine-utilizing transaminases represent a metabolic vulnerability in some breast tumor cells [42]. Furthermore, colorectal cancer cells harboring PI3KCA mutations like HCT116 cells display an increased expression of glutamate pyruvate transaminase 2 (GPT2) making them more dependent on glutamine [43]. In line with this, treatment with aminooxyacetate (AOA), a general inhibitor of transaminases, induced apoptosis in HCT-116 cells growing in glutamine-replete medium that was totally blocked in the presence of NEAA (Fig. 7A). To find out whether cell death induced by AOA was also dependent on GCN2 activation, we first examined whether the ISR was activated upon treatment of HCT116 cells with AOA. Results shown in figure S4A, left panel, revealed that addition of AOA to cultures of HCT116 cells activated a signaling pathway involving eiF2α phosphorylation and induction of ATF4 and CHOP transcription factors that was repressed in the presence of NEAA in the culture medium. Furthermore, GCN2 knockdown with two different siRNA oligonucleotides reduced AOA-induced eiF2α phosphorylation and CHOP induction (Fig. 7B). Importantly, GCN2 knockdown significantly inhibited AOA-induced apoptosis in HCT116 cells (Fig. 7C). Together, all these results suggest that similarly to the role of GCN2 in the activation of apoptosis in glutamine-starved cells, inhibiting NEAA synthesis by AOA triggers an apoptotic mechanism mediated by GCN2 activation of the ISR.

To get further insight into the mechanism underlying apoptosis induction upon NEAA limitation we examined the role of TRAIL-R2-regulated extrinsic apoptotic pathway by first analyzing TRAIL-R2 expression, FLIP levels and caspase-8 activation in HCT116 cells treated with AOA. Interestingly, AOA treatment led to a marked upregulation of TRAIL-R2 expression (Fig. S4A, right panel) and a decrease in FLIP levels (Fig. S4B) that were prevented by adding NEAA to the extracellular medium. Importantly, caspase-8 processing induced by AOA was clearly hindered in the presence of NEAA in the culture medium (Fig. S4C). Strikingly, silencing GCN2 expression abrogated AOA-induced TRAIL-R2 upregulation (Fig. 7D) which further supported a role of the extrinsic apoptotic pathway in GCN2-mediated cell death under conditions of NEAA limitation. To directly confirm the involvement of the extrinsic apoptotic pathway in the cell death response activated following NEAA limitation, TRAIL-R2 or caspase-8 expression was reduced by RNA interference prior to AOA treatment. As shown in Fig. 7E, either TRAIL-R2 or caspase-8 silencing markedly inhibited AOA-induced apoptosis in HCT116 cells up to 48 hours of treatment. All these results indicate that inhibition of transaminases in HCT116 colon cancer cells elicits an apoptotic response involving both FLIP_L_ downregulation and GCN2-mediated TRAIL-R2 upregulation, mainly due to impairment of NEAA biosynthesis.

## Discussion

Despite the fact that glutamine is a non-essential amino acid different types of cancer cells exhibit a marked dependence on glutamine for cell survival and proliferation [22, 43–47]. However, the precise mechanism underlying tumor cell death upon glutamine or NEAA limitation has not been fully elucidated. Our results demonstrate for the first time that inhibiting glutamine utilization in tumor cells with constitutive activation of the PI3Kinase/Akt pathway [43, 46], induces cell death by GCN2-mediated triggering of a TRAIL-R2/FADD/Caspase-8 apoptotic pathway that requires amplification through the mitochondria. Our results also reveal that in HCT116 Bax/Bak KO cells, incapable of triggering the mitochondrial apoptotic pathway and the feedback amplification of apical caspase activation, there is still caspase-8 activation upon glutamine deprivation. Together with the observation that silencing TRAIL-R2, FADD or Caspase-8 expression inhibits apoptosis induced upon glutamine limitation, all these findings point to a crucial role of the extrinsic pathway of apoptosis in tumor cell death under conditions of amino acids shortage.

Ligation of pro-apoptotic TRAIL receptors by their ligand TRAIL leads to death-inducing signaling complex (DISC) formation and caspase-8 activation to trigger the extrinsic pathway of apoptosis [48]. Our results demonstrate that when tumor cells are grown in the absence of glutamine, stimulation of the GCN2/ATF4/CHOP pathway results in TRAIL-R2 upregulation, an important event leading to caspase-8 activation and apoptosis. Interestingly, TRAIL-R2-dependent apoptotic response in glutamine-deprived tumor cells is ligand independent, as it has been described in cells facing other kind of stressful conditions [9–12]. Deciphering the cellular localization of TRAIL-R2 that results in caspase-8 activation and apoptosis in glutamine-deprived tumor cells is an issue that requires further investigation. One possible explanation for this observation is that accumulation of TRAIL-R2 either at the plasma membrane [49–52] or at intracellular membranes of the secretory pathway [9–11] could lead to oligomerization and auto-activation of the receptor. In this respect, TRAIL may be dispensable for TRAIL-R2 activation when receptor levels exceed a certain threshold, as reported for ectopic expression of TRAIL-R2 [52]. Indeed, ligand-independent assembly of the DISC has been demonstrated in the tumor necrosis factor (TNF) family of death receptors, most likely because of the homotypic association of receptors mediated by the pre-ligand-binding assembly domain [53]. Alternatively, oligomerization of a TRAIL-R2 transmembrane domain could also drive downstream apoptotic signaling [54].

Since the DISC components may colocalize in an intracellular membrane fraction in the absence of TRAIL [55], the increased expression of TRAIL-R2 and downregulation of cFLIP induced by glutamine deprivation in glutamine-addicted tumor cells could result in the formation of an intracellular DISC containing TRAIL-R2, FADD and procaspase-8 where caspase-8 is activated. Otherwise, it has been recently demonstrated that misfolded proteins can bind to and activate TRAIL-R2 at the ER-Golgi intermediate compartment [56]. Since glutamine deprivation can lead to improper protein folding [28], it is possible that receptor activation by misfolded proteins may take place under those circumstances. In addition, down-regulation of FLIP under glutamine deprivation can promote TRAIL-R2-mediated procaspase-8 oligomer assembly and cell death [40], according to the co-operative and hierarchical binding model [39].

**Fig. 8:**
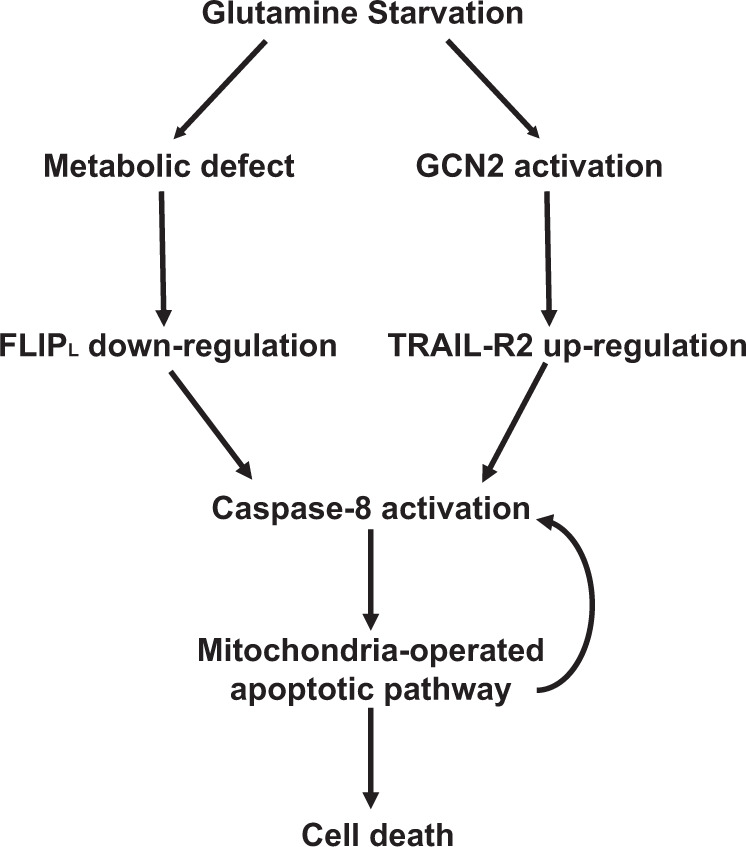
Schematic diagram of the apoptosis mechanism activated by glutamine limitation in glutamine-addicted tumor cells. Glutamine deprivation or inhibition of glutamine metabolism will result in GCN2-mediated TRAIL-R2 upregulation and metabolic stress-induced FLIP_L_ downregulation, leading to caspase-8 activation and apoptosis.

In summary, we have identified a signaling mechanism activated by the limitation in the availability of glutamine or its metabolism in glutamine-addicted tumor cells, which links GCN2/ISR activation with TRAIL-R2 upregulation. At the same time, metabolic stress resulting from the loss of cellular αKG will lead to a decrease of FLIP_L_ levels that in cooperation with TRAIL-R2 accumulation will result in caspase-8 activation and apoptosis (Fig. 8). Therefore, taking into account data from other studies, GCN2 activation may play a dual role in the regulation of tumor cell fate under metabolic stress. It will initially serve as a cell survival mechanism in starving tumor cells to restore homeostasis. However, under sustained or excessive stress, GCN2 will trigger a program of extrinsic apoptosis signaling to eliminate the damaged cells. Importantly, because glutamine levels are severely depleted in growing tumors, understanding how tumor cells scape from activation of this apoptosis mechanism is an essential question to address that may disclose new targets for therapeutic intervention.

## Materials and methods

### Cell culture

HEK293, HCT116 and HCT116 Bax/Bak K.O. cell lines were kindly provided by Dr. A. Rodriguez (Universidad Autónoma Madrid, Spain), Dr. J.A. Pintor-Toro (CABIMER, Seville, Spain) and Dr. C. Muñoz-Pinedo (Idibell, Barcelona, Spain), respectively. MDA-MB468 cells were a gift from Dr. J. Arribas (VallD’Hebron Institute of Oncology, Barcelona, Spain). MDA-MB468 and HEK293 cells were maintained in DMEM medium supplemented with 10% fetal bovine serum (Gibco), 2 mM L-glutamine, 50 U of penicillin/ml and 50 µg of streptomycin/ml. HCT116 and HCT116 Bax/Bak K.O. cells, in McCoy’s medium supplemented with 10% fetal bovine serum (Gibco), 2 mM L-glutamine, 50 U of penicillin/ml and 50 µg of streptomycin/ml. Cells were grown at 37 °C in a 5% CO_2_-humidified, 95% air incubator. All cell lines were regularly tested for mycoplasma contamination. Glutamine deprivation experiments were performed in DMEM medium lacking NEAA, with 10% dialyzed fetal bovine serum, penicillin (50 U/ml), and streptomycin (50 μg/ml). RPMI 1640 medium lacking methionine (Sigma-Aldrich) with 10% dialyzed fetal bovine serum, glutamine, penicillin and streptomycin was used in experiments to assess the response of tumor cells to methionine starvation.

### Reagents and antibodies

Media supplements and chemical reagents for molecular biology and buffer preparation were from Sigma-Aldrich, (St. Louis, MO, USA). Anti-Hsp70 (H5147) antibody, propidium iodide, dimethyl α-ketoglutarate, doxycycline and puromycin were obtained from Sigma-Aldrich. PE-IgG Isotype control (554680), anti-Bcl-xL (556361) and anti-procaspase-3 (C-31720) antibodies, were obtained from BD Biosciences (Erembodegem, Belgium). Anti-human PE-TRAIL-R2 antibody (307405) was from Biolegend (San Diego, USA). Anti-procaspase-8 antibody (04-574) was from Upstate Millipore (NY, USA). Anti-α-tubulin (SC-23948), anti-GAPDH (SC-47724), anti-GCN2 (SC-374609) and anti-ATF4 (D4B8) antibodies were from Santa Cruz Biotechnology (CA, USA). Anti-TRAIL-R1 (AF347), anti-TRAIL-R2 (AF631) and Recombinant TRAIL-R2-Fc chimeric protein (631-T2) were from R&D Systems (Minneapolis, USA). Anti-c-FLIP monoclonal antibody 7F10 (ALX-804-961-0100) was from Enzo Life Sciences (NY, USA). Anti-p-eIF2α (S51) (3597), anti-eIF2α (D7D3) (5324), anti-CHOP (D46F1) (5554), anti-caspase 8 (1C12) (9746), anti-cleaved caspase-8 (18C8) (9496), anti-cleaved caspase-9 (9501) and anti-cleaved caspase-3 (9661) antibodies were purchased from Cell Signaling Technology (CA, USA). Anti-proaspase-9 (M054-3) was from MBL (MA, USA). Horseradish peroxidase-conjugated secondary antibodies were obtained from DAKO (P0447, P0448, P0449) (Cambridge, UK). Recombinant human TRAIL was produced as previously described [57]. Q-VD-OPh was from AppexBio (Houston, USA). A92 was purchased from AXON Medchem (The Netherlands).

### Determination of apoptosis

Cells (3 × 10^5^/well) were treated in six-well plates as indicated in the figure legends. After treatment, hypodiploid apoptotic cells were detected by flow cytometry according to published procedures [55]. Quantitative analysis of the cell cycle and subG1 cells was carried out in a FACSCalibur cytometer using the Cell Quest software (Becton Dickinson, Mountain View, CA, USA).

### Analysis of cell viability by propidium iodide uptake

Cells (3 × 10^5^/well) were treated in 6-well plates as indicated in the figure legends. After treatment, cells are washed twice with PBS + 0.1% BSA, and incubated for 15 min on ice in the dark in the same buffer containing propidium iodide (2 μg/ml). Quantitative analysis of propidium iodide uptake was performed in a FACSCalibur cytometer using the Cell Quest Software (Becton Dickinson, Mountain View, CA, USA).

### Immunoblot analysis of proteins

Cells (3 × 10^5^) were washed with phosphate-buffered saline (PBS) and lysed in TR3 buffer (10 mM Na2HPO4, 10% Glycerol, 3% SDS). Protein content was measured with the Bradford reagent (Bio-Rad Laboratories, USA), before adding Laemmli sample buffer. Proteins were resolved on SDS-polyacrylamide minigels and detected as described previously [55]. Tubulin, β-Actin, Hsp70, and GAPDH were used as protein loading controls. Original western blots are included as Supplementary Material.

### Real time-qPCR

RNA was extracted using PRImeZOL (Canvax Biotech Córdoba, Spain) reagent, following the manufacturer’s instructions. mRNA expression was analyzed in triplicate by RT-qPCR on the ABI Prism7500 sequence detection system using predesigned Assay-on-demand primers and probes (Applied Biosystems). Hypoxanthine-guanine phosphoribosyltransferase (HPRT1 Hs01003267_m1) was used as an internal control and mRNA expression levels of TRAIL-R2 and TRAIL were given as fraction of mRNA levels in control cells. Primers and probes used were: TRAIL-R2 (TNFRSF1 Hs 00366278_m1) and TRAIL (TNFSF10 Hs00921974_m1).

### RNA interference

siRNAs against GCN2, caspase-8, FLIP_L_ and non-targeting scrambled oligonucleotide were synthesized by Sigma (St. Louis, MO). HCT116 cells were transfected with siRNAs using jetPRIME (Polyplus Transfection) following manufacturer instructions. After 24 h, transfection medium was replaced with regular medium and cells were incubated for 48 h before further analysis. For siRNA transfection in MDA-MB468 cells, Dharmafect-1 (Dharmacon) was used as described by the manufacturer. After 6 h, transfection medium was replaced with regular medium and cells were further incubated for 48 h before analysis.

### siRNAs

**Table:** 

Caspase-8:	5′-GUUCCUGAGCCUGGACUACdTdT-3′
GCN2#1	5′-CAGCAGAAAUCAUGUACGAdTdT-3′
GCN2#2:	5′-CACCGUCAAGAUUACGGACUAdTdT-3′
FLIP_L_#4	5′-CCUAGGAAUCUGCCUGAUAdTdT-3′
Scrambled:	5′-CUUUGGGUGAUCUACGUUAdTdT-3′

### Analysis of TRAIL receptors by flow cytometry

Cells were detached with trypsin solution and re-suspended in growth media. After incubation for 15 min under cell culture conditions (37 °C in a 5% CO_2_-humidified, 95% air incubator), cells were washed with ice-cold phosphate-buffered saline (PBS) and re-suspended in PBS. Cells were then labeled either with 5 µg/ml of anti-TRAIL-R2-PE, or an IgG-PE control antibody (BD Bioscience) for 30 min on ice and darkness. Analysis of the receptor cell surface expression was carried out in a FACSCalibur cytometer using the Cell Quest Software (Becton Dickinson, Mountain View, CA, USA).

### Generation of HCT116 and MDA-MB468 cell lines

FLIP(L), dnFADD and Bcl-xL retroviral vectors for stable gene expression have been described previously [55, 58]. For silencing experiments, shRNAs against GCN2, caspase-8, TRAILR2, TRAIL in a pSUPER vector (OligoEngine) were digested and cloned between *EcoR1 and Cla1* into a H1 promoter-driven GFP-encoding pLVTHM lentiviral vector [59]. Lentiviruses and retroviruses were produced by transfection of HEK293-T cells by the calcium phosphate method with the corresponding vectors. Lentivirus or retrovirus-containing supernatants were collected 48 h after transfection and concentrated by ultracentrifugation at 22,000 rpm for 90 min at 4 °C.

### shRNAs sequences

**Table:** 

GCN2:	5′-GATCCCC**CAGCAGAAATCATGTACGA**TTCAAGAGA**TCGTACATGATTTCTGCTG**TTTTTA-3′
TRAIL-R2#1:	5′-GATCCCC**GACCCTTGTGCTCGTTGTC**TTCAAGAGA**GACAACGAGCACAAGGGTCT**TTTTTA-3′
TRAIL-R2#2:	5′-GATCCCC**TCATGTATCTAGAAGGTAA**TTCAAGAGA**TTACCTTCTAGATACATGA**TTTTTA-3′
TRAIL:	5′-GATCCCC**GCAGCTCACATAACTGGGA**TTCAAGAGA**TCCCAGTTATGTGAGCTGC**TTTTTA-3′
Caspase-8:	5′-GATCCCC**GGAGCTGCTCTTCCGAATT**TTCAAGAGA**AATTCGGAAGAGCAGCTCC**TTTTTA-3′
Scrambled:	5′-GATCCCC**CTTTGGGTGATCTACGTTA**TTCAAGAGA**TAACGTAGATCACCCAAAG**TTTTTA-3′

Stable populations of HCT116 and MDA-MB468 cell lines infected with retroviruses were obtained after selection in culture medium containing puromycin (1.5 µg/ml) during 48 h. Tumor cells infected with GFP-expressing lentiviruses were detected by flow cytometry.

HCT116 and MDA-MB468 cells stably overexpressing human TRAILR-2 were generated by lentiviral transduction of full length human *TRAIL-R2* cloned into pCW57-MCS1-P2A-MCS2 (a kind donation of Dr. Markus Rehm, University of Stuttgart, Germany) as reported [60]. Cells transduced with empty vectors served as controls. TRAIL-R2 expression was induced using 1 µg/ml doxycycline (Sigma-Aldrich, St. Louis, Missouri, USA).

### Statistical analysis

All data are presented as the mean ± standard deviation (SD) of at least three independent experiments. Statistical analysis was performed using GraphPAD Prism 9 (GraphPad Software, San Diego, CA, USA). The differences among different groups were determined by the two-way ANOVA, Tukey’s multiple comparison test. *P* < 0.05 was considered significant. n.s. non-significant; *P < 0.05; **P < 0.01; ***P < 0.001; ****P < 0.0001.

## Supplementary information

Supplementary figure legends

Figure S1

Figure S2

Figure S3

Figure S4

Original Data File

Reproducibility checklist

## Data Availability

All data generated or analyzed during this study are included in the main text and the supplementary information files.

## References

[1] Ashkenazi A, Dixit VM (1998). Death receptors: signaling and modulation. Science.

[2] Wiley SR, Schooley K, Smolak PJ, Din WS, Huang CP, Nicholl JK (1995). Identification and characterization of a new member of the TNF family that induces apoptosis. Immunity.

[3] Ashkenazi A, Pai RC, Fong S, Leung S, Lawrence DA, Marsters SA (1999). Safety and antitumor activity of recombinant soluble Apo2 ligand. J Clin Invest.

[4] Walczak H, Miller RE, Ariail K, Gliniak B, Griffith TS, Kubin M (1999). Tumoricidal activity of tumor necrosis factor-related apoptosis-inducing ligand in vivo. Nat Med.

[5] Sprick MR, Weigand MA, Rieser E, Rauch CT, Juo P, Blenis J (2000). FADD/MORT1 and caspase-8 are recruited to TRAIL receptors 1 and 2 and are essential for apoptosis mediated by TRAIL receptor 2. Immunity.

[6] Irmler M, Thome M, Hahne M, Schneider P, Hofmann K, Steiner V (1997). Inhibition of death receptor signals by cellular FLIP. Nature.

[7] Humphreys LM, Fox JP, Higgins CA, Majkut J, Sessler T, McLaughlin K (2020). A revised model of TRAIL-R2 DISC assembly explains how FLIP(L) can inhibit or promote apoptosis. EMBO Rep..

[8] Kline CL, Van den Heuvel AP, Allen JE, Prabhu VV, Dicker DT, El-Deiry WS (2016). ONC201 kills solid tumor cells by triggering an integrated stress response dependent on ATF4 activation by specific eIF2alpha kinases. Sci Signal.

[9] Lu M, Lawrence DA, Marsters S, Acosta-Alvear D, Kimmig P, Mendez AS (2014). Opposing unfolded-protein-response signals converge on death receptor 5 to control apoptosis. Science.

[10] Martin-Perez R, Palacios C, Yerbes R, Cano-Gonzalez A, Iglesias-Serret D, Gil J (2014). Activated ERBB2/HER2 licenses sensitivity to apoptosis upon endoplasmic reticulum stress through a PERK-dependent pathway. Cancer Res.

[11] Martin-Perez R, Yerbes R, Mora-Molina R, Cano-Gonzalez A, Arribas J, Mazzone M (2017). Oncogenic p95HER2/611CTF primes human breast epithelial cells for metabolic stress-induced down-regulation of FLIP and activation of TRAIL-R/Caspase-8-dependent apoptosis. Oncotarget.

[12] Iurlaro R, Puschel F, Leon-Annicchiarico CL, O'Connor H, Martin SJ, Palou-Gramon D (2017). Glucose deprivation induces ATF4-mediated apoptosis through TRAIL death receptors. Mol Cell Biol.

[13] DeBerardinis RJ, Cheng T (2010). Q’s next: the diverse functions of glutamine in metabolism, cell biology and cancer. Oncogene.

[14] Vaupel P, Kallinowski F, Okunieff P (1989). Blood flow, oxygen and nutrient supply, and metabolic microenvironment of human tumors: a review. Cancer Res.

[15] Kamphorst JJ, Nofal M, Commisso C, Hackett SR, Lu W, Grabocka E (2015). Human pancreatic cancer tumors are nutrient poor and tumor cells actively scavenge extracellular protein. Cancer Res.

[16] Pan M, Reid MA, Lowman XH, Kulkarni RP, Tran TQ, Liu X (2016). Regional glutamine deficiency in tumours promotes dedifferentiation through inhibition of histone demethylation. Nat Cell Biol.

[17] Kilberg MS, Pan YX, Chen H, Leung-Pineda V (2005). Nutritional control of gene expression: how mammalian cells respond to amino acid limitation. Annu Rev Nutr.

[18] Harding HP, Novoa I, Zhang Y, Zeng H, Wek R, Schapira M (2000). Regulated translation initiation controls stress-induced gene expression in mammalian cells. Mol Cell.

[19] Misra J, Holmes MJ, E TM, Langevin M, Kim HG, Carlson KR (2021). Discordant regulation of eIF2 kinase GCN2 and mTORC1 during nutrient stress. Nucleic Acids Res.

[20] Schmidt S, Gay D, Uthe FW, Denk S, Paauwe M, Matthes N (2019). A MYC-GCN2-eIF2alpha negative feedback loop limits protein synthesis to prevent MYC-dependent apoptosis in colorectal cancer. Nat Cell Biol.

[21] Ye J, Kumanova M, Hart LS, Sloane K, Zhang H, De Panis DN (2010). The GCN2-ATF4 pathway is critical for tumour cell survival and proliferation in response to nutrient deprivation. EMBO J.

[22] Qing G, Li B, Vu A, Skuli N, Walton ZE, Liu X (2012). ATF4 regulates MYC-mediated neuroblastoma cell death upon glutamine deprivation. Cancer Cell.

[23] Shi WZ, Tian Y, Li J (2019). GCN2 suppression attenuates cerebral ischemia in mice by reducing apoptosis and endoplasmic reticulum (ER) stress through the blockage of FoxO3a-regulated ROS production. Biochem Biophys Res Commun.

[24] Wei C, Lin M, Jinjun B, Su F, Dan C, Yan C (2015). Involvement of general control nonderepressible kinase 2 in cancer cell apoptosis by posttranslational mechanisms. Mol Biol Cell.

[25] Zhang X, He N, Xing Y, Lu Y (2020). Knockdown of GCN2 inhibits high glucose-induced oxidative stress and apoptosis in retinal pigment epithelial cells. Clin Exp Pharm Physiol.

[26] Jones RG, Thompson CB (2009). Tumor suppressors and cell metabolism: a recipe for cancer growth. Genes Dev.

[27] Momcilovic M, Bailey ST, Lee JT, Fishbein MC, Braas D, Go J (2018). The GSK3 signaling axis regulates adaptive glutamine metabolism in lung squamous cell carcinoma. Cancer Cell.

[28] Altman BJ, Stine ZE, Dang CV (2016). From Krebs to clinic: glutamine metabolism to cancer therapy. Nat Rev Cancer.

[29] Pakos-Zebrucka K, Koryga I, Mnich K, Ljujic M, Samali A, Gorman AM (2016). The integrated stress response. EMBO Rep..

[30] Zinszner H, Kuroda M, Wang X, Batchvarova N, Lightfoot RT, Remotti H (1998). CHOP is implicated in programmed cell death in response to impaired function of the endoplasmic reticulum. Genes Dev.

[31] Harding HP, Zhang Y, Zeng H, Novoa I, Lu PD, Calfon M (2003). An integrated stress response regulates amino acid metabolism and resistance to oxidative stress. Mol Cell.

[32] Inglis AJ, Masson GR, Shao S, Perisic O, McLaughlin SH, Hegde RS (2019). Activation of GCN2 by the ribosomal P-stalk. Proc Natl Acad Sci USA.

[33] Ishimura R, Nagy G, Dotu I, Chuang JH, Ackerman SL (2016). Activation of GCN2 kinase by ribosome stalling links translation elongation with translation initiation. Elife.

[34] Mauro-Lizcano M, Lopez-Rivas A (2018). Glutamine metabolism regulates FLIP expression and sensitivity to TRAIL in triple-negative breast cancer cells. Cell Death Dis.

[35] Yamaguchi H, Wang HG (2004). CHOP is involved in endoplasmic reticulum stress-induced apoptosis by enhancing DR5 expression in human carcinoma cells. J Biol Chem.

[36] Villar VH, Nguyen TL, Delcroix V, Teres S, Bouchecareilh M, Salin B (2017). mTORC1 inhibition in cancer cells protects from glutaminolysis-mediated apoptosis during nutrient limitation. Nat Commun.

[37] Mills KR, Reginato M, Debnath J, Queenan B, Brugge JS (2004). Tumor necrosis factor-related apoptosis-inducing ligand (TRAIL) is required for induction of autophagy during lumen formation in vitro. Proc Natl Acad Sci USA.

[38] McComb S, Chan PK, Guinot A, Hartmannsdottir H, Jenni S, Dobay MP (2019). Efficient apoptosis requires feedback amplification of upstream apoptotic signals by effector caspase-3 or -7. Sci Adv.

[39] Hughes MA, Powley IR, Jukes-Jones R, Horn S, Feoktistova M, Fairall L (2016). Co-operative and hierarchical binding of c-FLIP and caspase-8: a unified model defines how c-FLIP isoforms differentially control cell fate. Mol Cell.

[40] Wilson TR, McLaughlin KM, McEwan M, Sakai H, Rogers KM, Redmond KM (2007). c-FLIP: a key regulator of colorectal cancer cell death. Cancer Res.

[41] Coloff JL, Murphy JP, Braun CR, Harris IS, Shelton LM, Kami K (2016). Differential glutamate metabolism in proliferating and quiescent mammary epithelial cells. Cell Metab.

[42] Yang CS, Stampouloglou E, Kingston NM, Zhang L, Monti S, Varelas X (2018). Glutamine-utilizing transaminases are a metabolic vulnerability of TAZ/YAP-activated cancer cells. EMBO Rep.

[43] Hao Y, Samuels Y, Li Q, Krokowski D, Guan BJ, Wang C (2016). Oncogenic PIK3CA mutations reprogram glutamine metabolism in colorectal cancer. Nat Commun.

[44] Cetinbas NM, Sudderth J, Harris RC, Cebeci A, Negri GL, Yilmaz OH (2016). Glucose-dependent anaplerosis in cancer cells is required for cellular redox balance in the absence of glutamine. Sci Rep..

[45] Kung HN, Marks JR, Chi JT (2011). Glutamine synthetase is a genetic determinant of cell type-specific glutamine independence in breast epithelia. PLoS Genet.

[46] Morotti M, Zois CE, El-Ansari R, Craze ML, Rakha EA, Fan SJ (2021). Increased expression of glutamine transporter SNAT2/SLC38A2 promotes glutamine dependence and oxidative stress resistance, and is associated with worse prognosis in triple-negative breast cancer. Br J Cancer.

[47] Timmerman LA, Holton T, Yuneva M, Louie RJ, Padro M, Daemen A (2013). Glutamine sensitivity analysis identifies the xCT antiporter as a common triple-negative breast tumor therapeutic target. Cancer Cell.

[48] Kischkel FC, Lawrence DA, Chuntharapai A, Schow P, Kim KJ, Ashkenazi A (2000). Apo2L/TRAIL-dependent recruitment of endogenous FADD and caspase-8 to death receptors 4 and 5. Immunity.

[49] Cazanave SC, Mott JL, Bronk SF, Werneburg NW, Fingas CD, Meng XW (2011). Death receptor 5 signaling promotes hepatocyte lipoapoptosis. J Biol Chem.

[50] Han B, Yao W, Oh YT, Tong JS, Li S, Deng J (2015). The novel proteasome inhibitor carfilzomib activates and enhances extrinsic apoptosis involving stabilization of death receptor 5. Oncotarget.

[51] Maldonado-Celis ME, Bousserouel S, Gosse F, Lobstein A, Raul F (2009). Apple procyanidins activate apoptotic signaling pathway in human colon adenocarcinoma cells by a lipid-raft independent mechanism. Biochem Biophys Res Commun.

[52] Sheridan JP, Marsters SA, Pitti RM, Gurney A, Skubatch M, Baldwin D (1997). Control of TRAIL-induced apoptosis by a family of signaling and decoy receptors. Science.

[53] Chan FK, Chun HJ, Zheng L, Siegel RM, Bui KL, Lenardo MJ (2000). A domain in TNF receptors that mediates ligand-independent receptor assembly and signaling. Science.

[54] Pan L, Fu TM, Zhao W, Zhao L, Chen W, Qiu C (2019). Higher-order clustering of the transmembrane anchor of DR5 drives signaling. Cell.

[55] Yerbes R, Palacios C, Reginato MJ, Lopez-Rivas A (2011). Cellular FLIP(L) plays a survival role and regulates morphogenesis in breast epithelial cells. Biochim Biophys Acta.

[56] Lam M, Marsters SA, Ashkenazi A, Walter P (2020). Misfolded proteins bind and activate death receptor 5 to trigger apoptosis during unresolved endoplasmic reticulum stress. Elife.

[57] Harper N, Farrow SN, Kaptein A, Cohen GM, MacFarlane M (2001). Modulation of tumor necrosis factor apoptosis-inducing ligand- induced NF-kappa B activation by inhibition of apical caspases. J Biol Chem.

[58] Caro-Maldonado A, Tait SW, Ramirez-Peinado S, Ricci JE, Fabregat I, Green DR (2010). Glucose deprivation induces an atypical form of apoptosis mediated by caspase-8 in Bax-, Bak-deficient cells. Cell Death Differ.

[59] Wiznerowicz M, Trono D (2003). Conditional suppression of cellular genes: lentivirus vector-mediated drug-inducible RNA interference. J Virol.

[60] Hagenlocher C, Siebert R, Taschke B, Wieske S, Hausser A, Rehm M (2022). ER stress-induced cell death proceeds independently of the TRAIL-R2 signaling axis in pancreatic beta cells. Cell Death Discov.

