# Supplementary material for: Limiting glutamine utilization activates a GCN2/TRAIL-R2/Caspase-8 apoptotic pathway in glutamine-addicted tumor cells
Source: Cell Death Dis. 2022 Oct 27;13(10):906. doi: 10.1038/s41419-022-05346-y (PMC9613879; doi:10.1038/s41419-022-05346-y)
Supplement: Supplementary file 1 — Supplementary figure legends [file 41419_2022_5346_MOESM1_ESM.docx]

**Supplementary figure legends**

**Figure S1 – Role of GCN2 and the TRAIL system in glutamine deprivation-induced apoptosis in tumor cells. A** MDA-MB468 cells were transfected with either a scrambled oligonucleotide or a siRNA targeting GCN2. 30 hours after transfection, cells were incubated for 72 h in complete or glutamine-depleted medium and cell death was determined by propidium iodide uptake (left panel). To analyze TRAIL-R2, GCN2 and CHOP protein levels by Western blotting, 30 hours after transfection, cells were further incubated for 24 h in complete or glutamine-depleted medium (right panel). Tubulin was used as protein-loading control. **B** HCT116 cells stably expressing scrambled or TRAIL-R2 targeting shRNA (shTR2#2) were incubated in medium with or without glutamine and apoptosis was assessed at 48 hours. TRAIL-R2 knockdown was determined by Western blotting. **C** Apoptosis was assessed in HCT116 cells incubated for 24 h in complete medium with or without TRAIL (50 ng/mL) in the presence or absence of 250 ng/ml of TRAIL-R2 Fc (left panel). In **C** (right panel) apoptosis was determined in HCT116 cells cultured for 24 h in medium with or without glutamine, in the presence or absence of TRAIL-R2 Fc (250 ng/ml). **D** MDA-MB468 cells stably expressing a scrambled (shSc) or a TRAIL targeting shRNA (shTRAIL) were cultured in the presence or absence of glutamine and apoptosis was measured at 48 h (left panel). TRAIL knockdown was assessed by RT-qPCR (right panel). **E** HCT116 cells were transfected either with a scrambled oligonucleotide or with a siRNA targeting caspase-8 (siC8) with a different sequence from the one used to generate shC8. 48 hours after transfection cells were incubated for 24 h in complete or glutamine-depleted medium and apoptosis was assessed. Data are presented as mean ± SD from at least three independent experiments. ns= non-significant; ***P*<0.01; *****P*<0. 0001; two-way ANOVA test. Tukey’s multiple comparison test.

**Figure S2. Methionine deprivation in glutamine-containing medium** **activates a GCN2-dependent TRAIL-R2 increase and apoptosis in HCT116 cells. A** HCT116 cells were transfected for 48 h with either a scrambled oligonucleotide (SC) or a siRNA targeting GCN2 (siGCN#1). After transfection, cells were incubated for 3 or 16 hours in complete or methionine-depleted medium. Levels of eIF2α phosphorylation, eIF2α, GCN2, ATF4, CHOP and TRAIL-R2 were assessed by Western blotting. Tubulin and HSP70 were used as protein-loading controls. **B** HCT116 cells were transfected for 48 h with either a scrambled oligonucleotide (SC) or siRNAs targeting GCN2 (siGCN#1), TRAIL-R2 (TR2#1) or Caspase-8 (Caspase-8#1). After transfection, cells were incubated for 16h in complete or methionine-depleted medium and apoptosis assessed as described in Materials and Methods section. Levels of GCN2, TRAIL-R2 and Caspase-8 were assessed by Western blotting. Tubulin was used as a protein-loading control.

**Figure S3. Ectopic overexpression of TRAIL-R2 co-operates with FLIP_L_ knockdown to induce caspase-8 activation and apoptosis in glutamine-addicted tumor cells.** HCT116 cells stably expressing either a scrambled or a GCN2 targeting shRNA were cultured in the presence or absence of glutamine for the indicated times and GCN2, FLIP_L_ or FLIP_S_ levels were assessed by Western blotting (**A**, left panel). HCT116 cells were cultured in the presence or absence of glutamine with or without 1 µM A92 for the indicated times. FLIP levels, as well as eIF2α phosphorylation and eIF2α levels were assessed by Western Blotting (**A**, right panel). **B** HCT116 (upper panel) or MDA-MB468 (lower panel) cells expressing doxycycline-inducible human TRAIL-R2 or control cells (pCW57-Φ), were stimulated with 1 µg/mL of doxycycline. TRAIL-R2 expression was monitored by immunoblotting after 24h (HCT116) or 30 h (MDA-MB468), and apoptosis after 48 h of doxycycline treatment. Data are presented as mean ± SD from at least three independent experiments. **C** HCT116 cells stably expressing doxycycline-inducible TRAIL-R2 or an empty plasmid were transfected for 48 h with either a scrambled oligonucleotide (Sc) or a siRNA targeting FLIP_L_ (siFLIP_L_#4). After transfection, cells were treated or not with doxycycline (1 µg/mL) to induce the expression of TRAIL-R2. Levels of FLIP_L_, FLIP_S_, TRAIL-R2 and processing of caspase-8 were assessed by Western blotting after 16 h of doxycycline treatment (left panel). Apoptosis was assessed as described in Materials and Methods section after 24 h of doxycycline treatment (right panel). Data are presented as mean ± SD from three independent experiments. ns = non-significant; **P*<0.05; ***P*<0.01; ****P*<0.001; two-way ANOVA text. Tukey’s multiple comparison test. **D** MDA-MB468 cells expressing doxycycline-inducible human TRAIL-R2 (pCW57-TR2) or control cells (pCW57-Φ) were transfected with either a scrambled oligonucleotide or a siRNA targeting FLIP_L_ (#4). 24 hours after transfection, cells were incubated with 1 µg/mL of doxycycline during 48 h to measure apoptosis. n.s. non significant; **P*<0.05; ***P*<0.01; ****P*<0.001; *****P*<0. 0001; two-way ANOVA test. Tukey’s multiple comparison test.

**Figure S4 –** **NEAA inhibit the ISR, TRAIL-R2 up-regulation, FLIP down-regulation and caspase-8 activation in tumor cells treated with AOA.** HCT116 cells were treated for 17 h with or without 1 mM AOA, in the presence or absence of NEAA. Following treatment, ISR activation and TRAIL-R2 levels (**A**), FLIP down-regulation (**B**) and caspase-8 activation (**C**) were analyzed by Western blotting with the indicated antibodies. GAPDH and Tubulin were used as protein-loading controls.
