# Supplementary material for: Limiting glutamine utilization activates a GCN2/TRAIL-R2/Caspase-8 apoptotic pathway in glutamine-addicted tumor cells
Source: Cell Death Dis. 2022 Oct 27;13(10):906. doi: 10.1038/s41419-022-05346-y (PMC9613879; doi:10.1038/s41419-022-05346-y)
Supplement: Supplementary file 6 — Original Data File [file 41419_2022_5346_MOESM6_ESM.pdf]

Fig. 1B

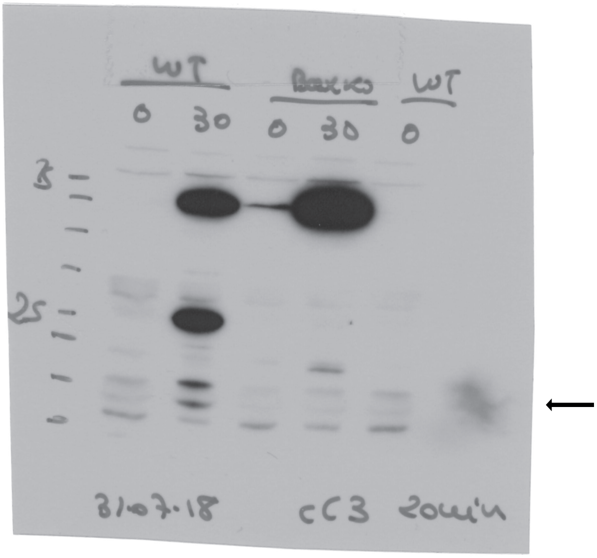

cC3

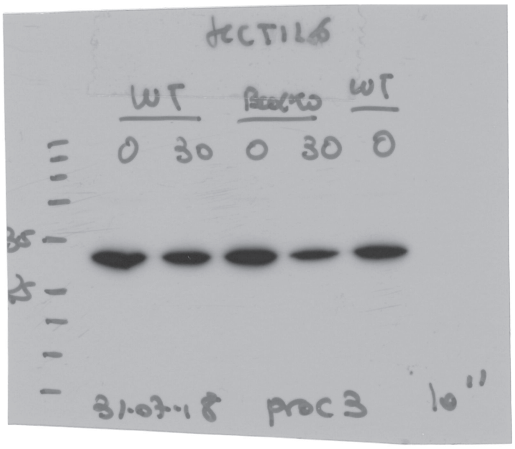

ProC3

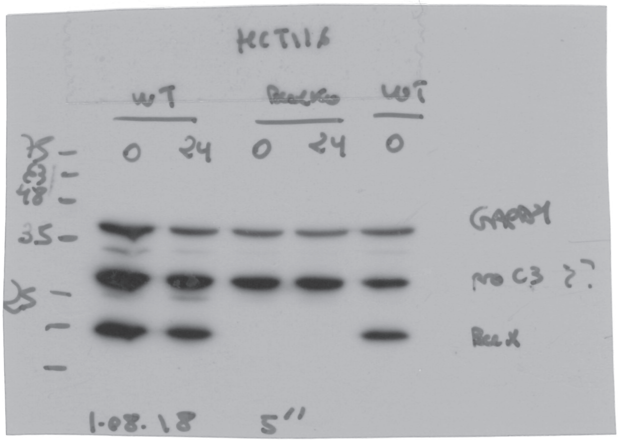

GAPDH

Fig. 1C

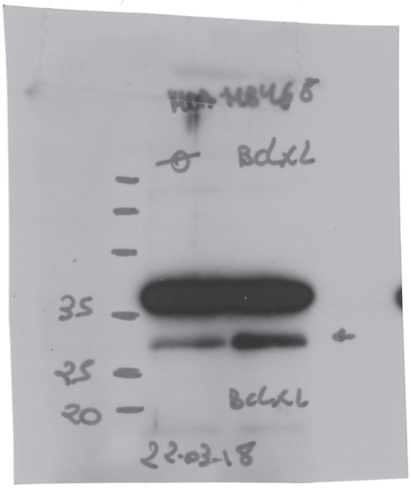

Bcl-Xl

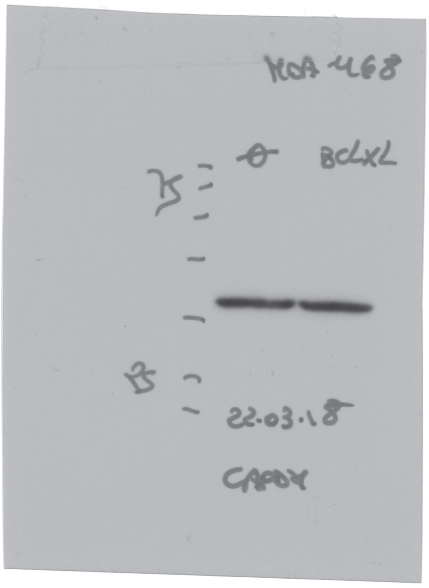

GAPDH

Figure 2A: eIF2 alpha

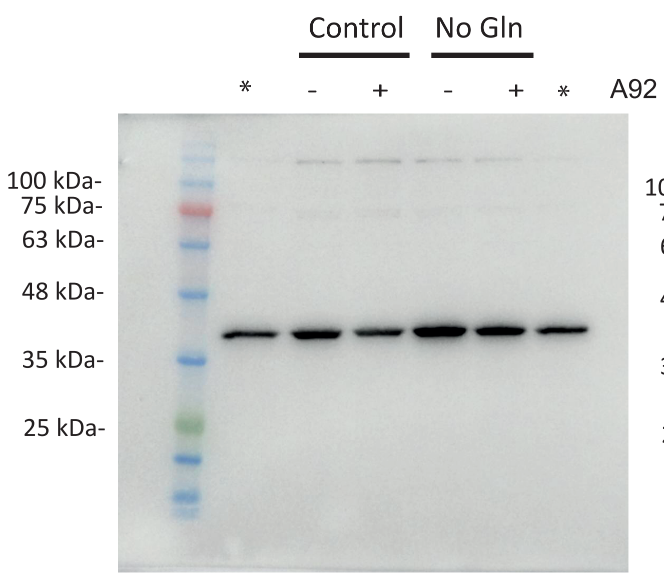

p-eIF2 alpha

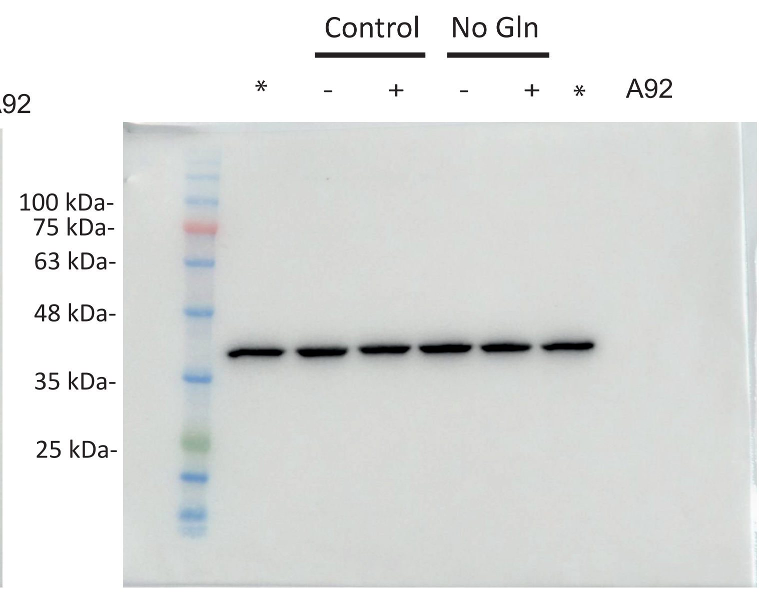

eIF2 alpha

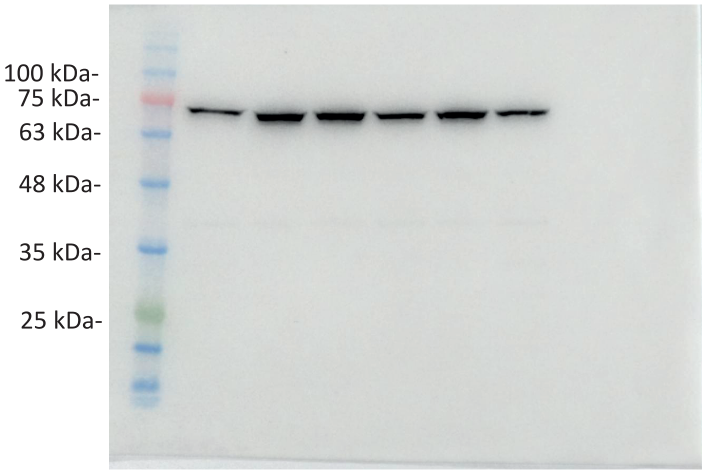

Hsp70

*Irrelevant sample

Figure 2A: ATF4_CHOP

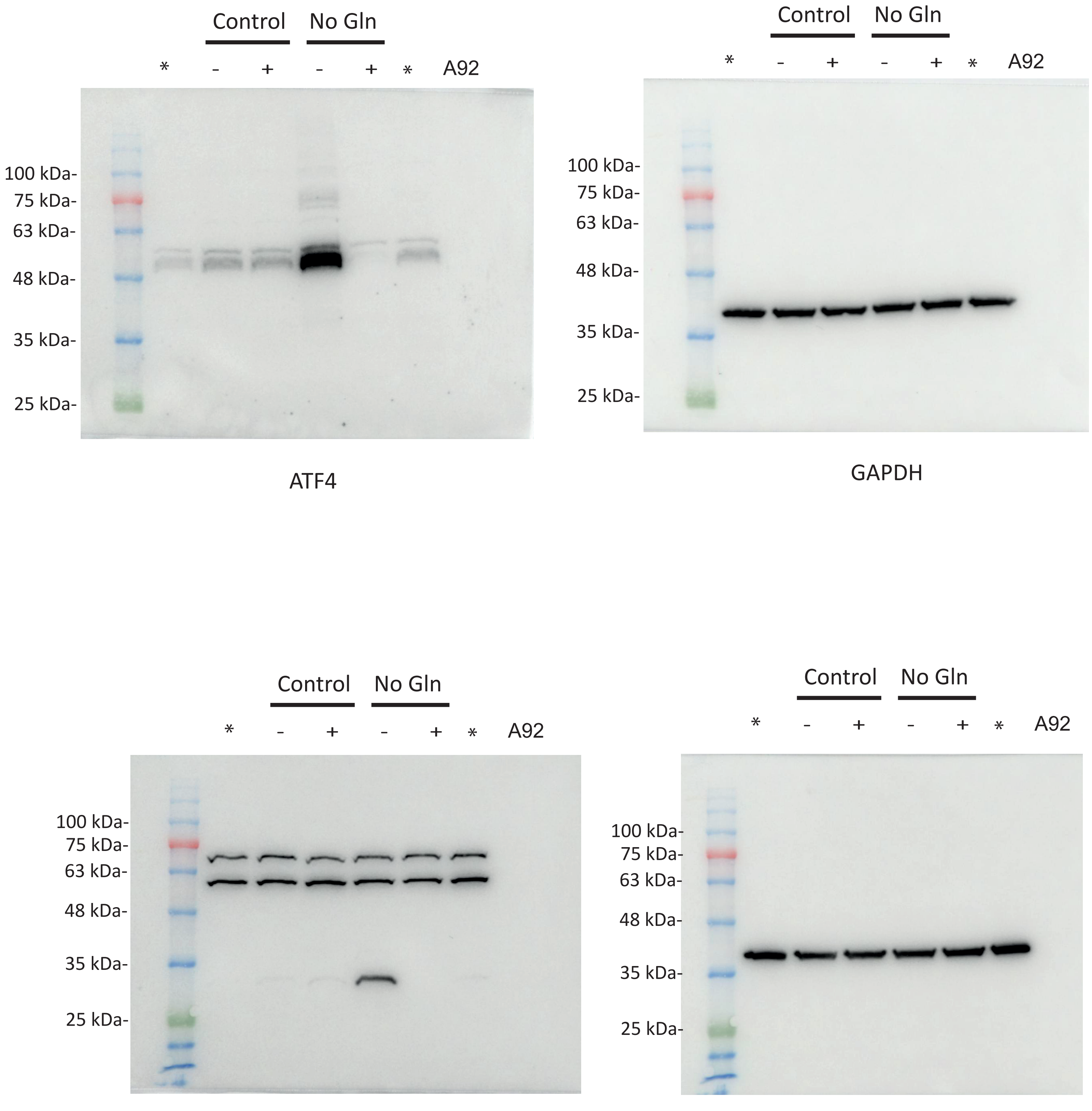

CHOP

GAPDH

*Irrelevant sample

Figure 2B left panel

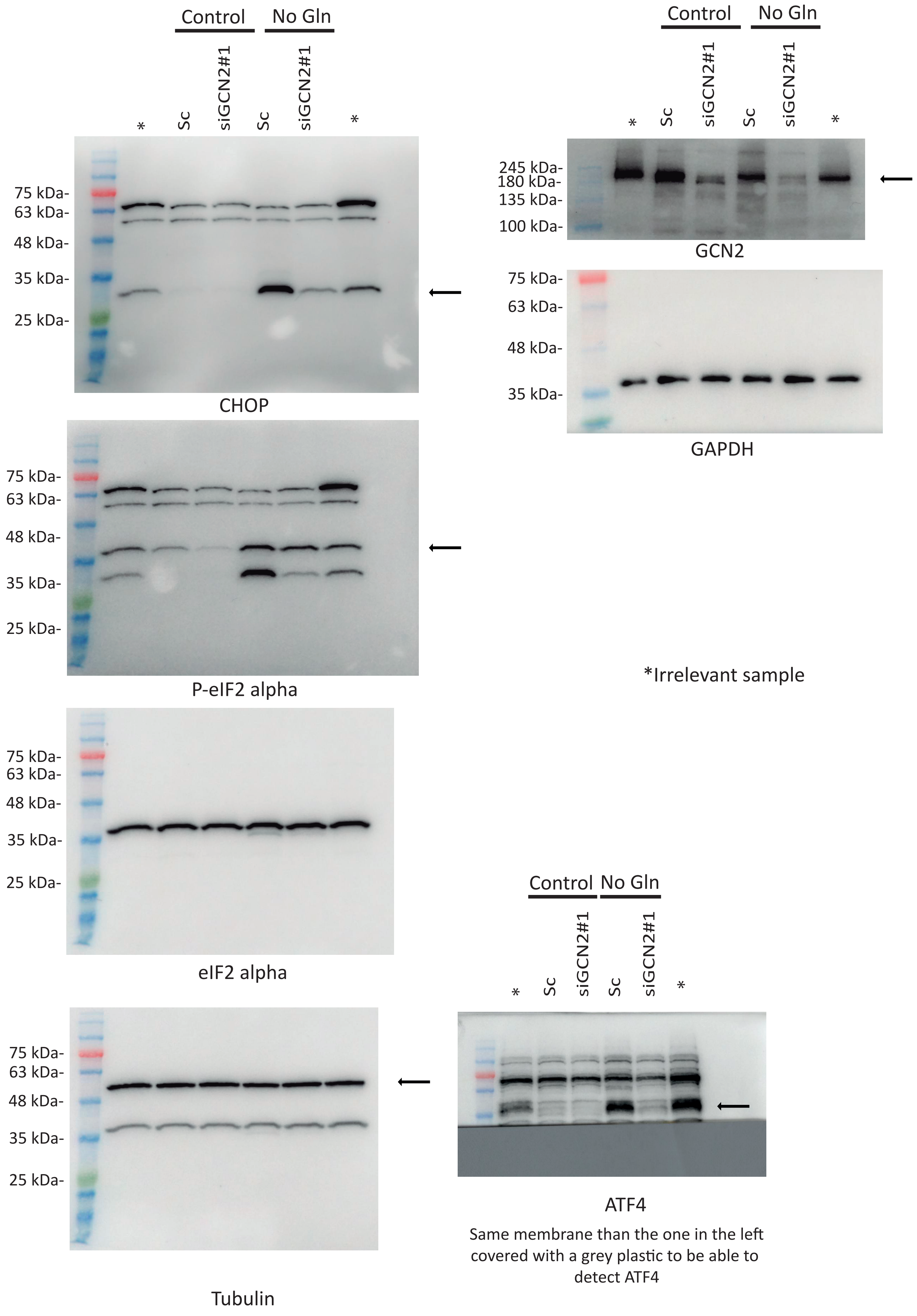

Figure 2B right panel

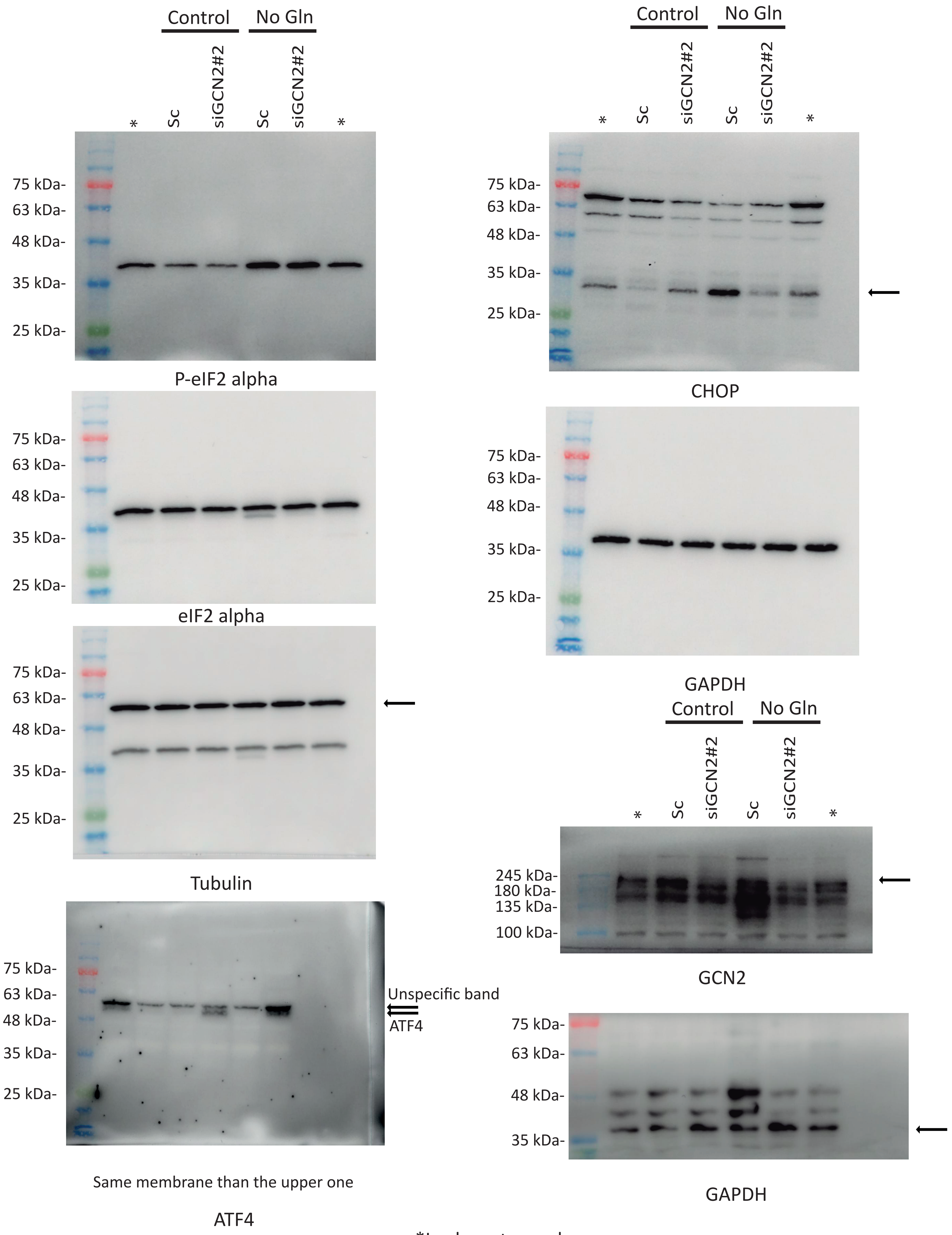

*Irrelevant sample

Fig. 3A

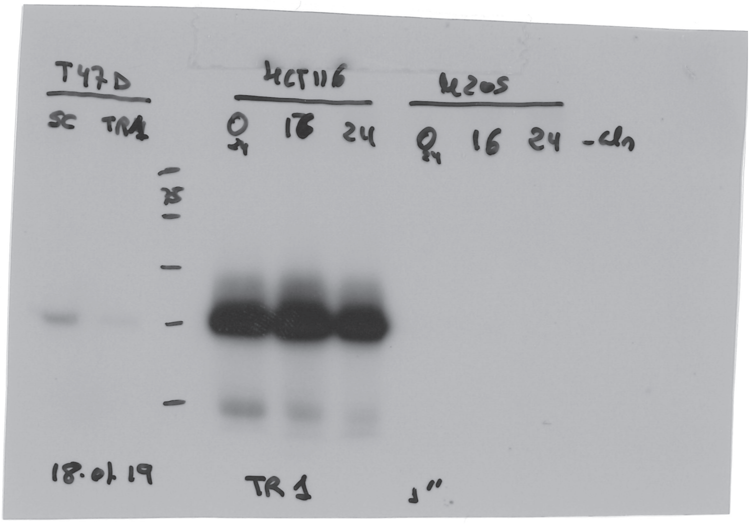

TRAIL-R1

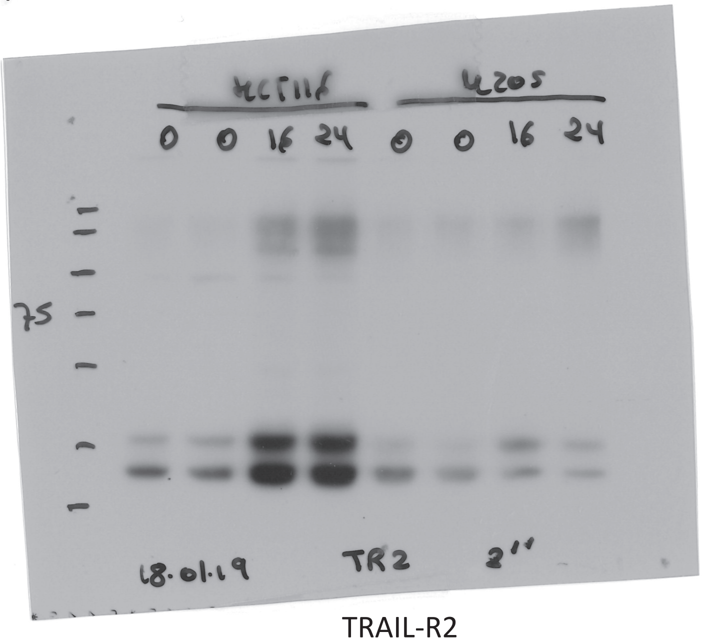

TRAIL-R2

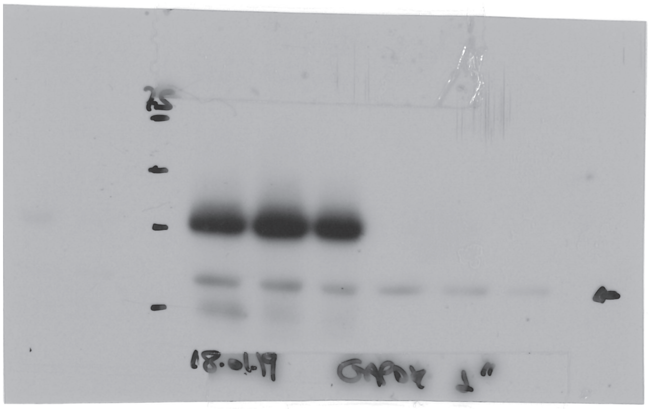

GAPDH

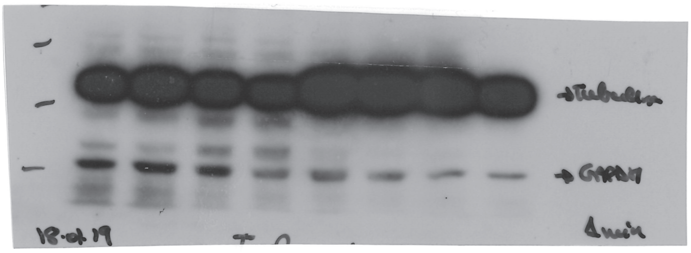

GAPDH

Fig. 3B

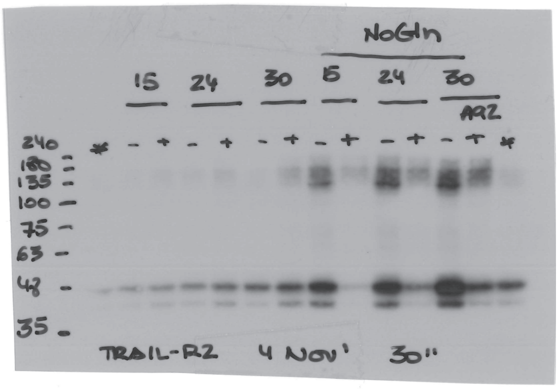

TRAIL-R2

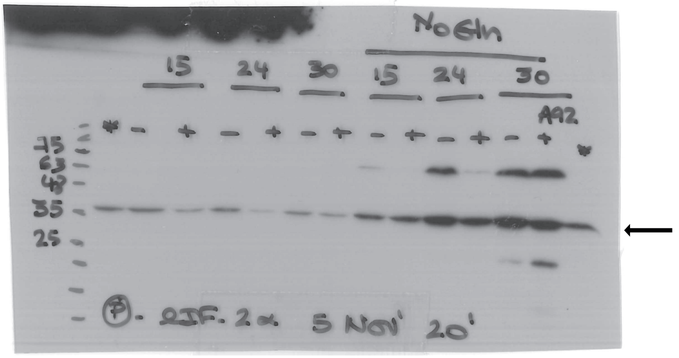

P-eIF2 alpha

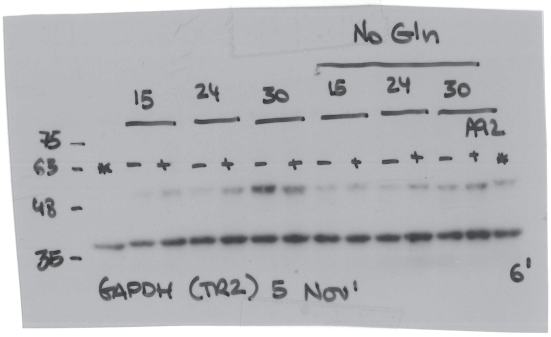

GAPDH

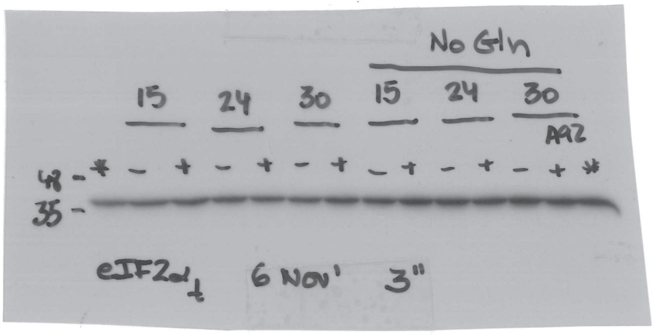

eIF2 alpha

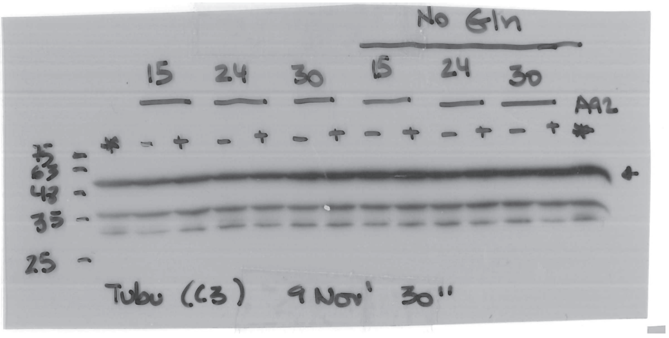

Tubulin

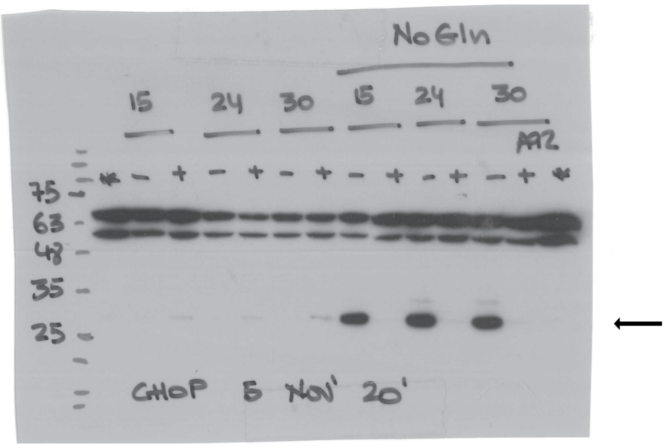

CHOP

*Irrelevant sample

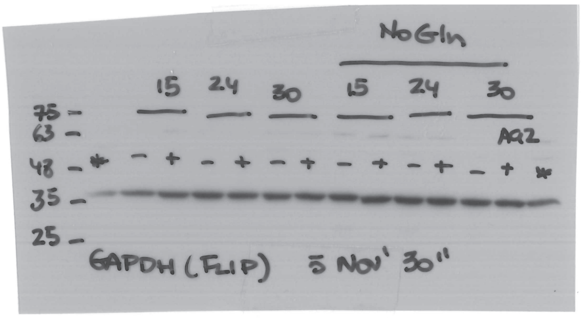

GAPDH

Fig. 3C

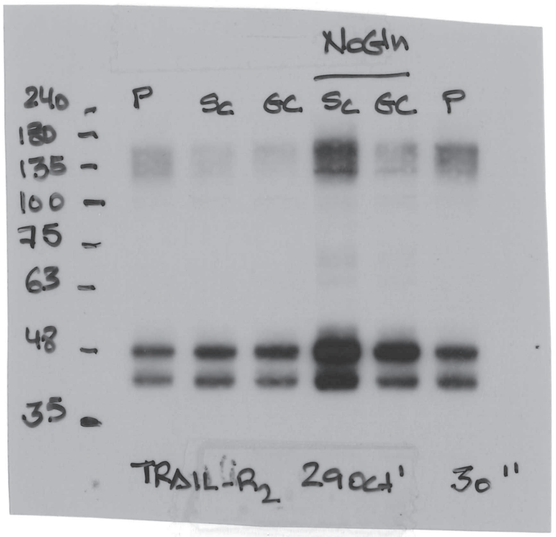

TRAIL-R2

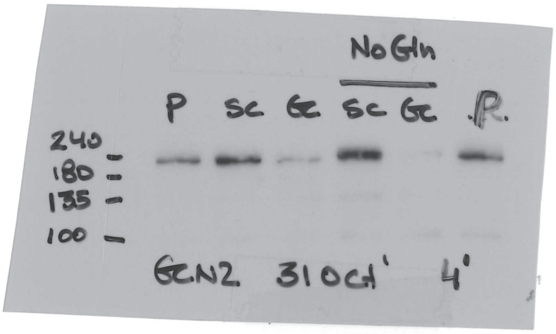

GCN2

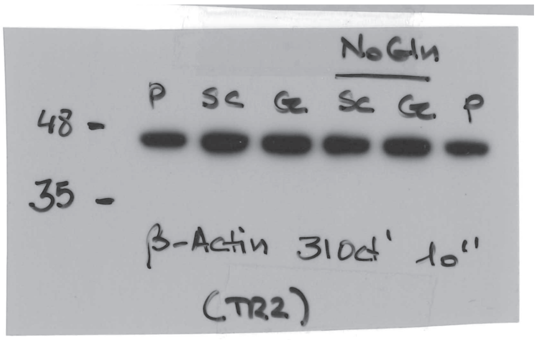

Beta-Actin

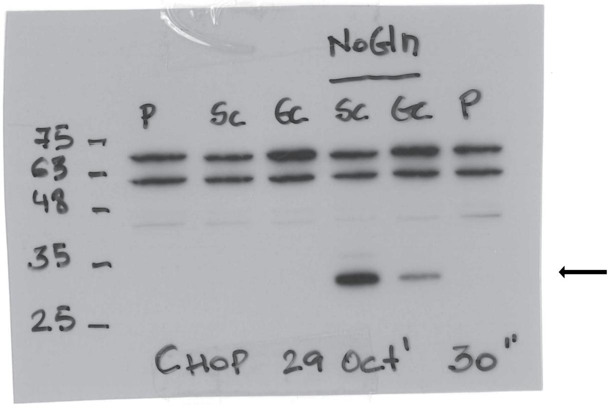

CHOP

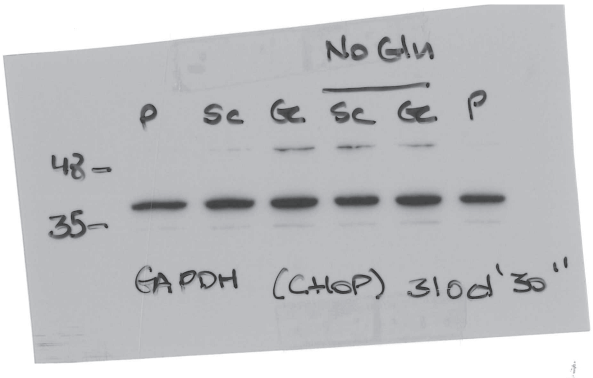

GAPDH

P: Irrelevant sample

Fig. 4A

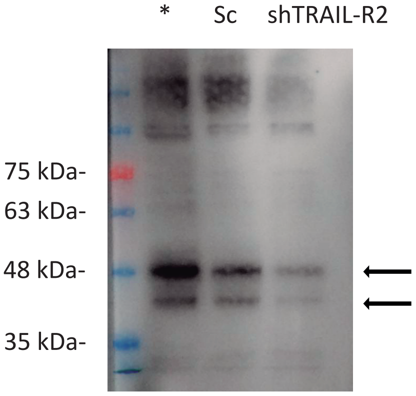

TRAIL-R2

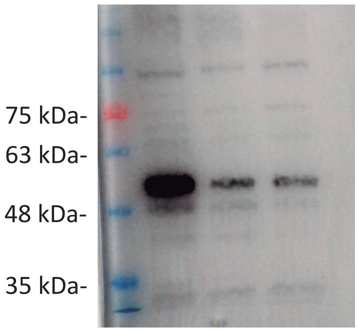

Tubulin

*Irrelevant sample

Fig. 4B

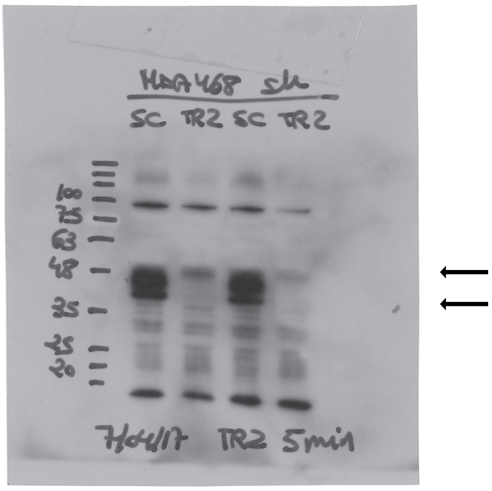

TRAIL-R2

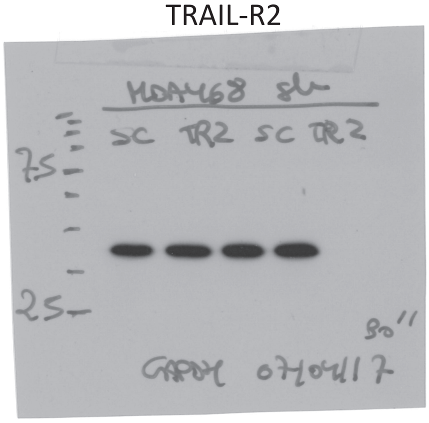

GAPDH

Figure 4C

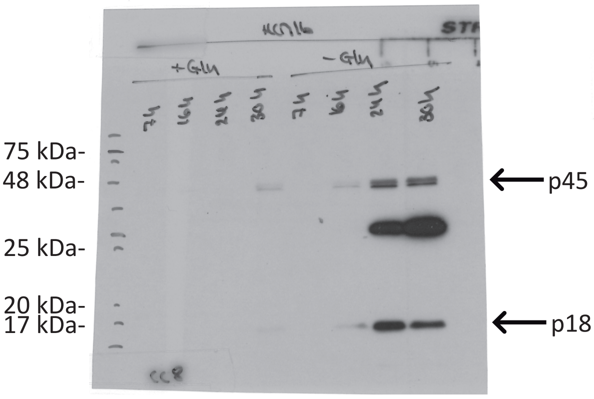

cC8

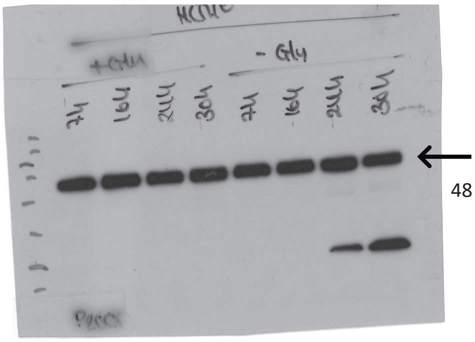

proC8

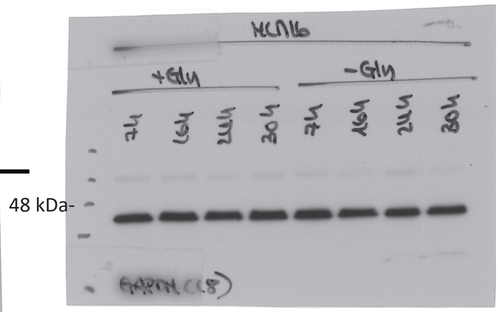

GAPDH

Fig. 4D

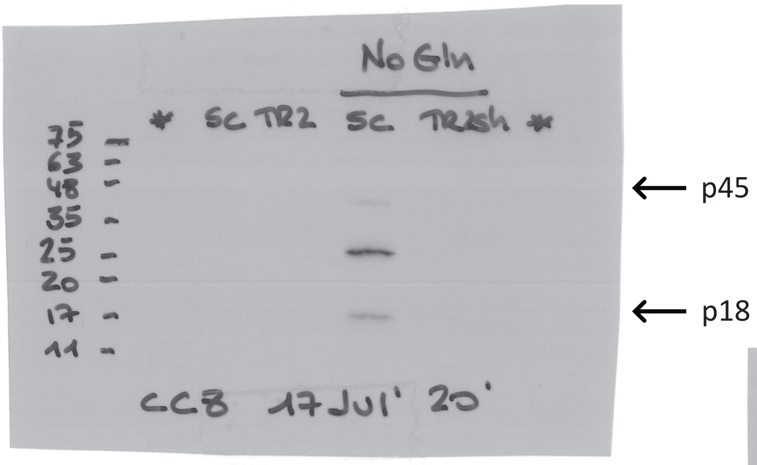

cC8

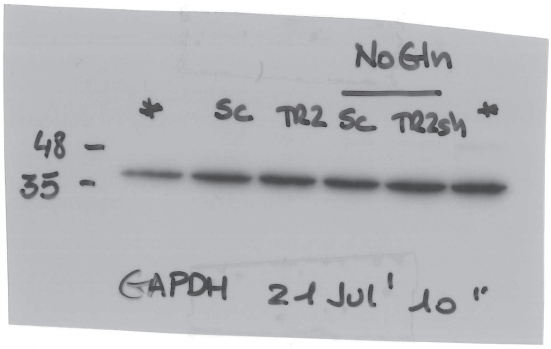

GAPDH

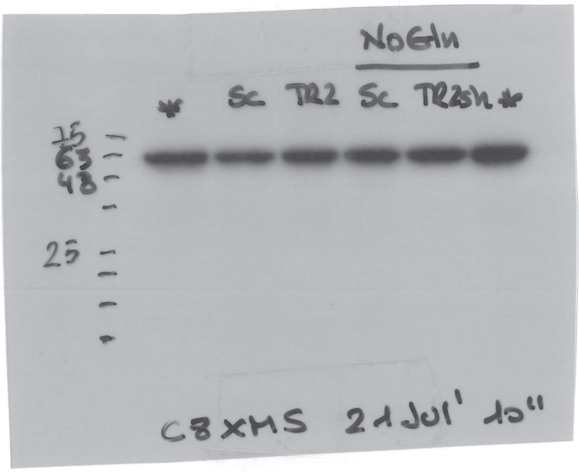

ProC8

*Irrelevant sample

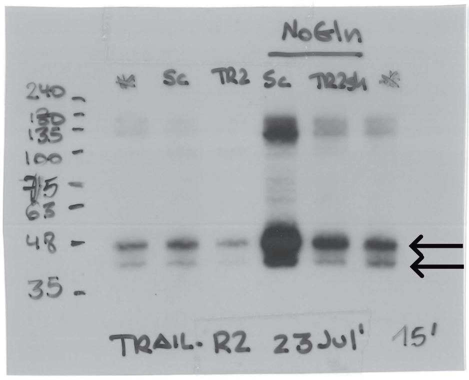

TRAIL-R2

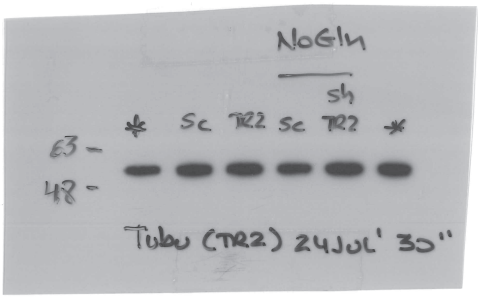

Tubulin

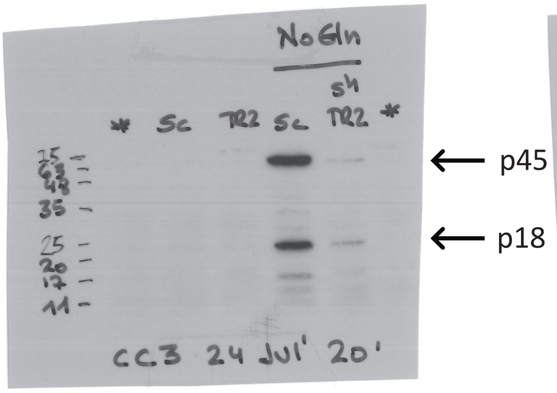

cC3

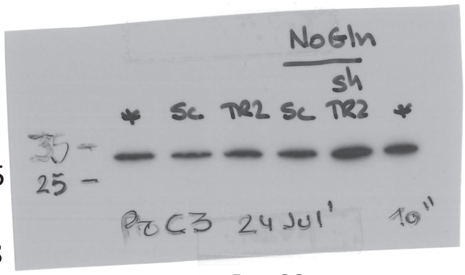

ProC3

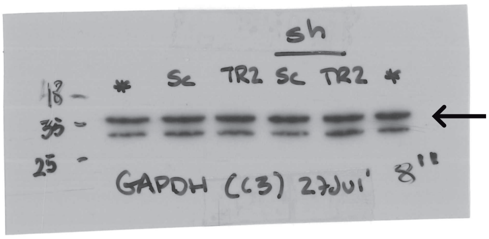

GAPDH

Fig. 5A

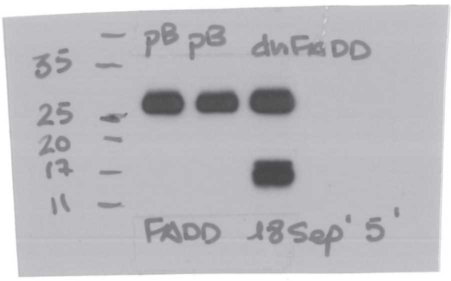

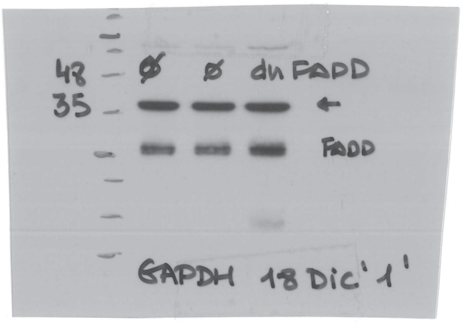

Fig. 5B upper

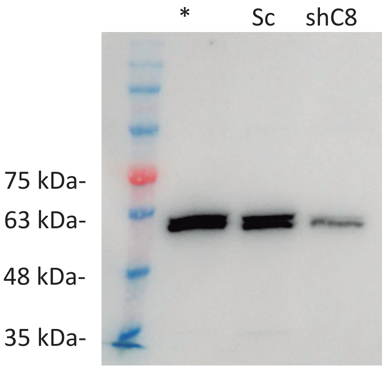

*Irrelevant sample

C8 XMS

Fig. 5B lower

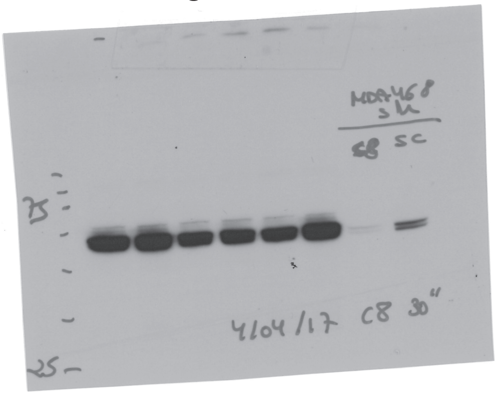

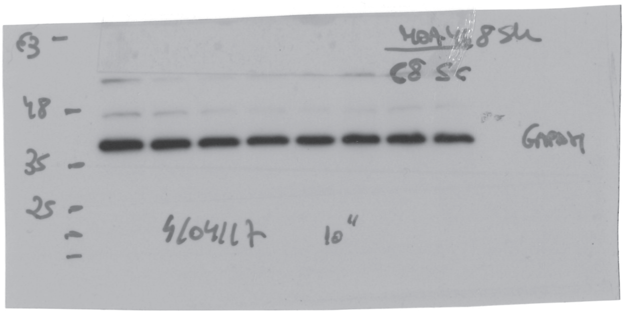

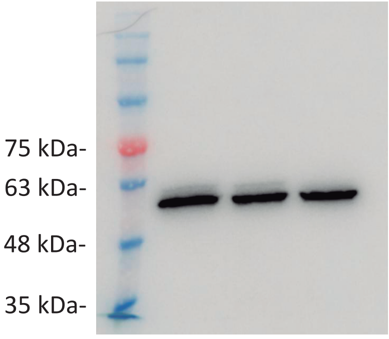

Tubulin

Fig. 5C

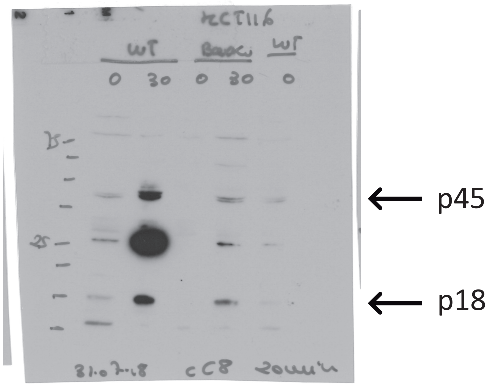

cC8

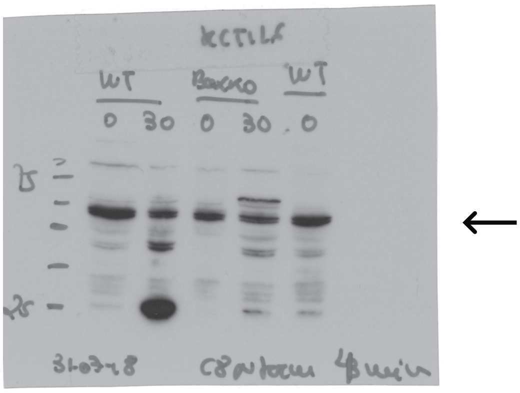

ProC8

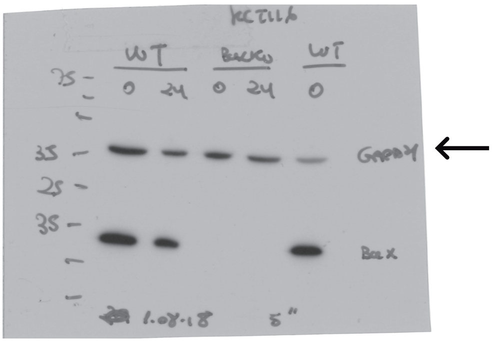

GAPDH

Figure 5D upper panel

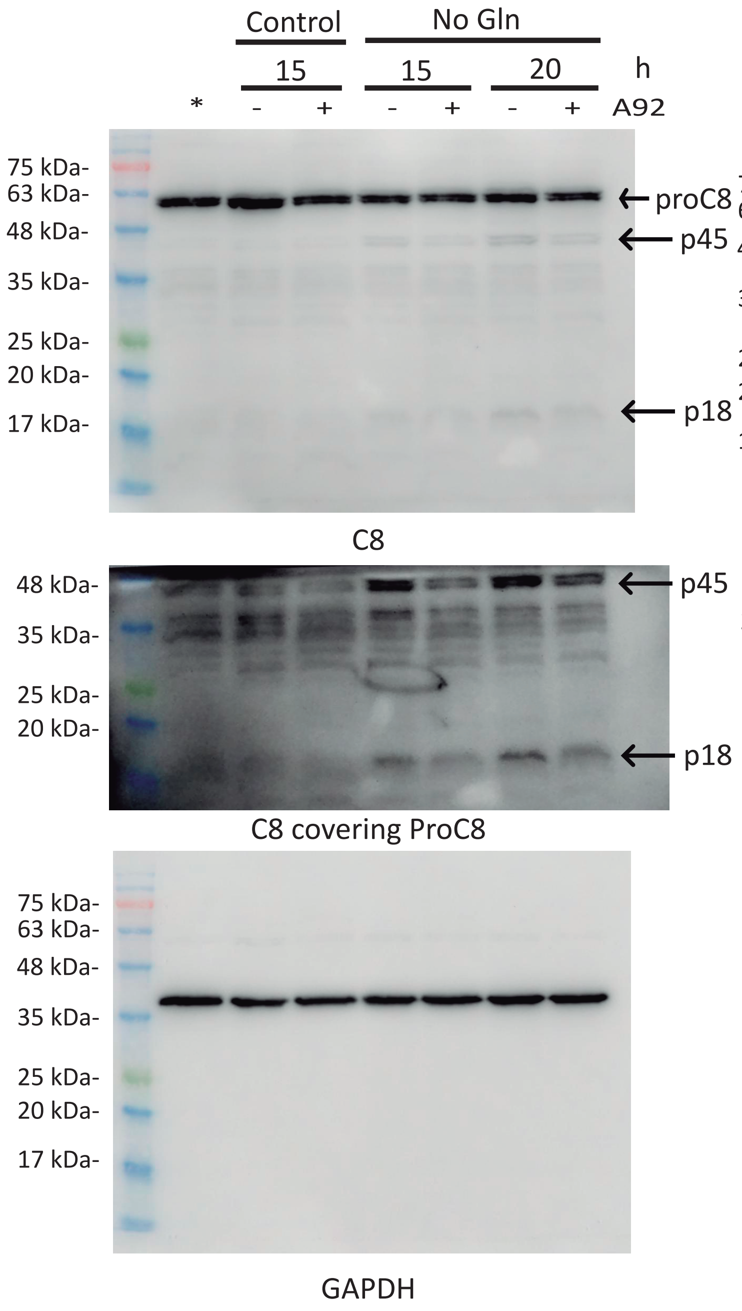

Figure 5D lower panel

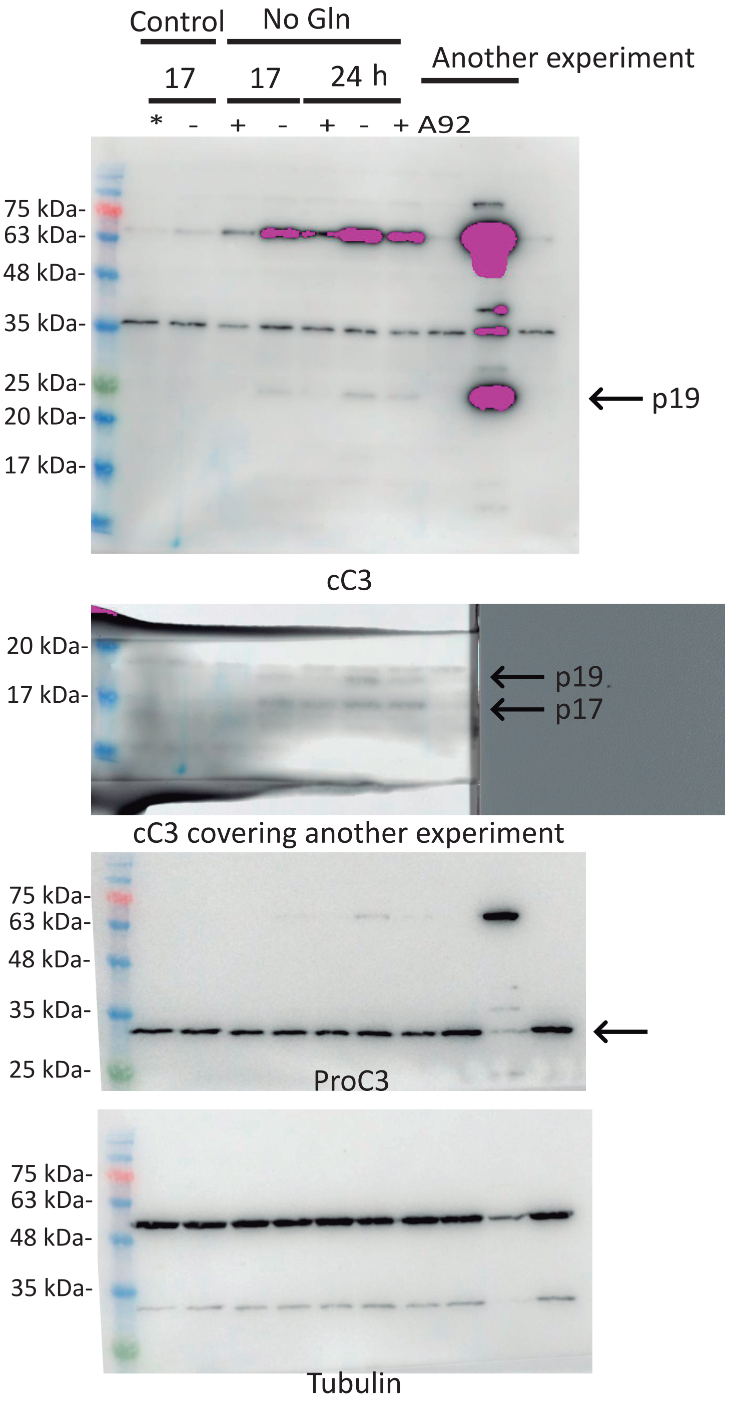

*Irrelevant sample

Fig. 5E

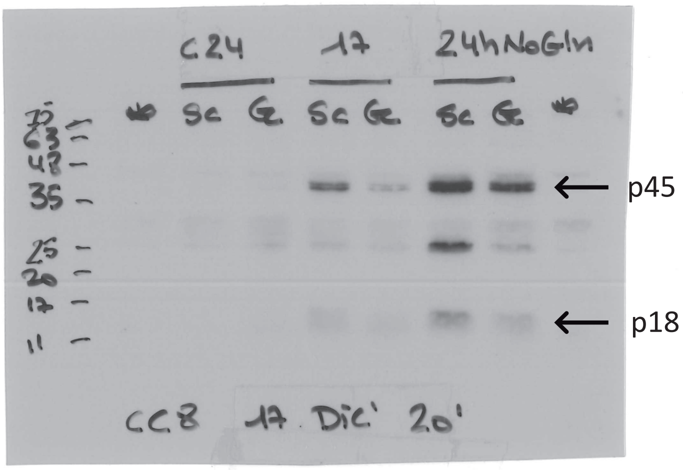

cC8

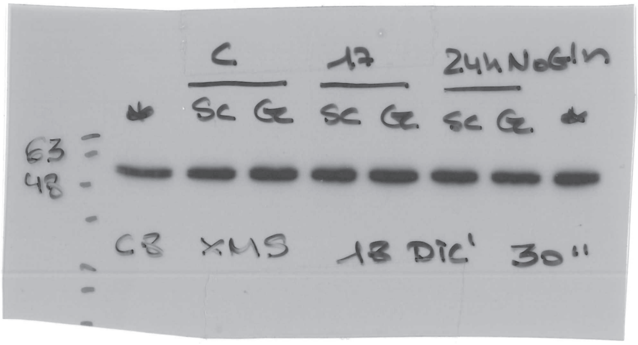

ProC8

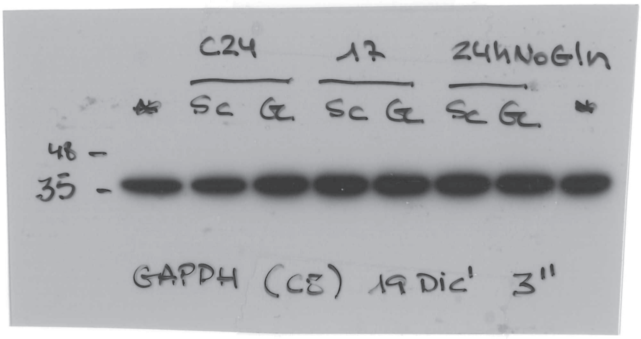

GAPDH

*Irrelevant sample

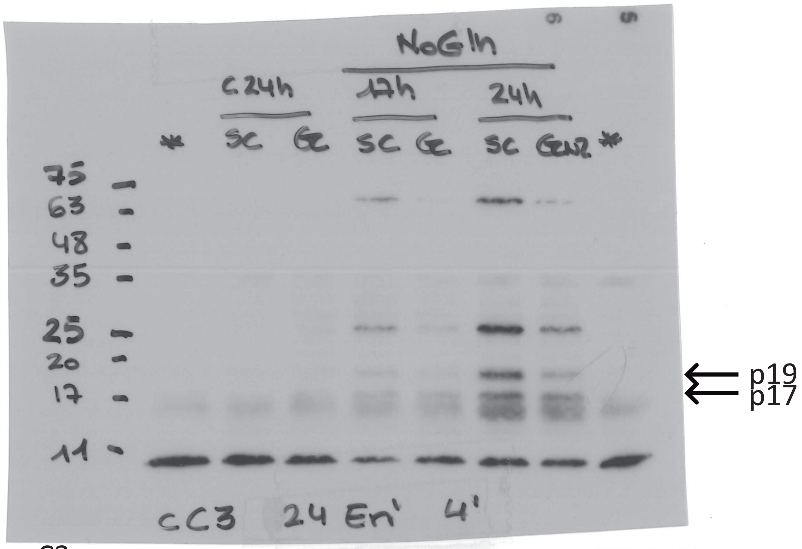

cC3

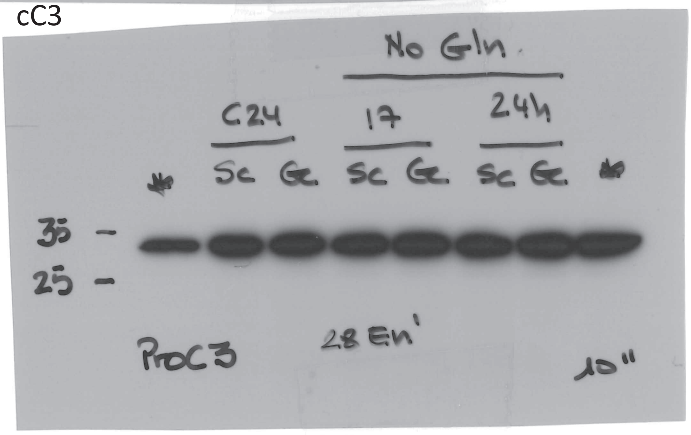

ProC3

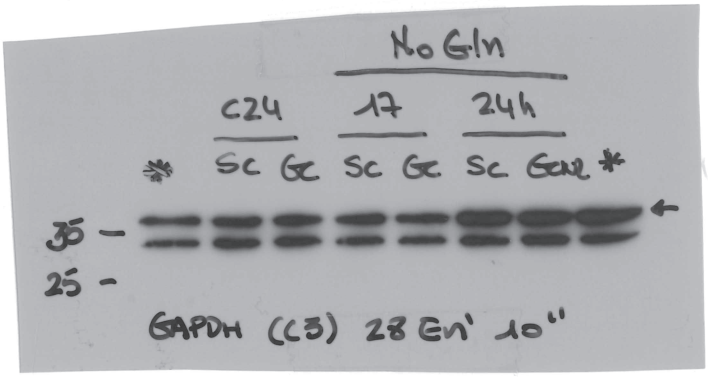

GAPDH

Figure 6A left panel

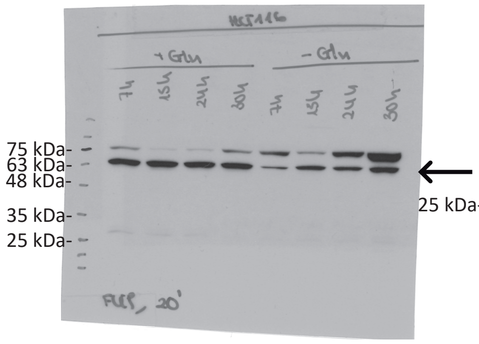

FLIPL

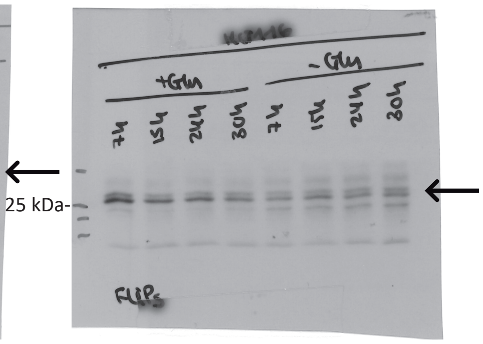

FLIPS

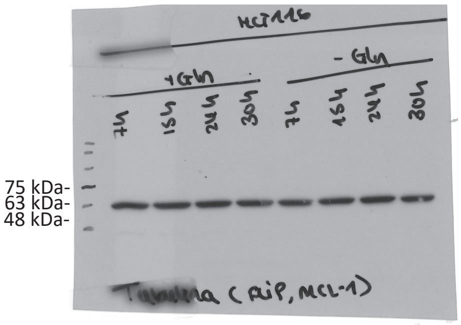

Tubulin

Figure 6A right panel

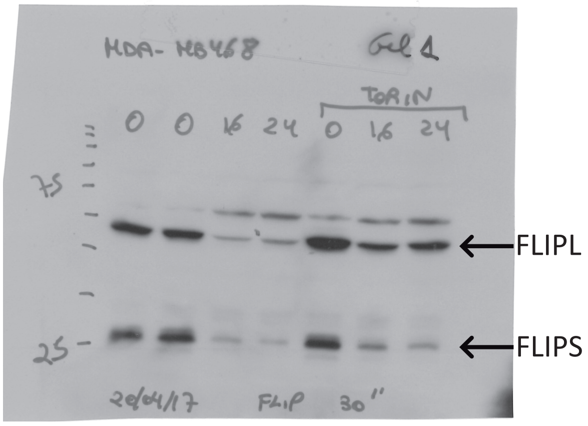

FLIP

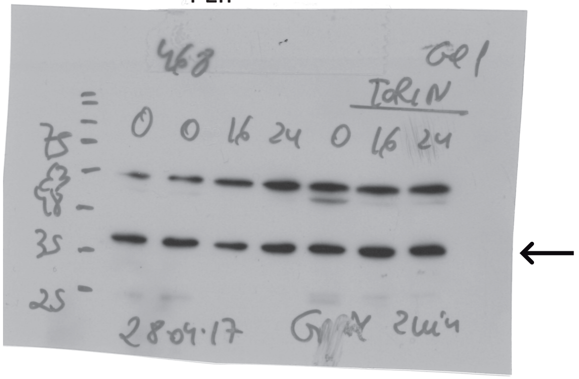

GAPDH

Figure 6B

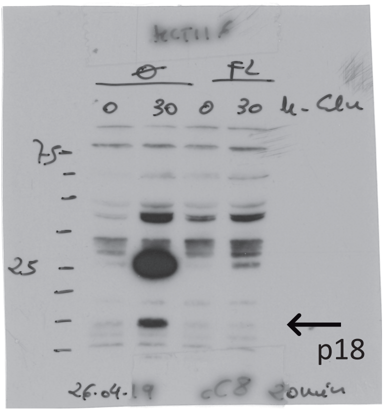

cC8

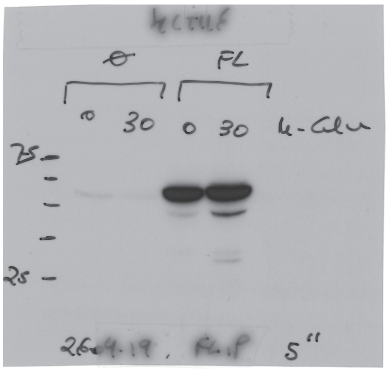

FLIP

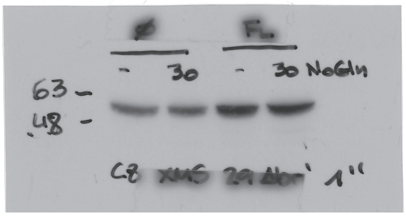

ProC8

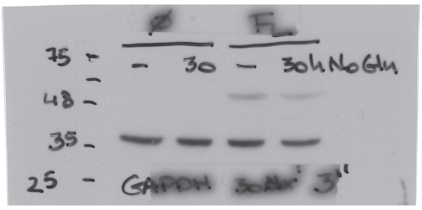

GAPDH

Figure 6C

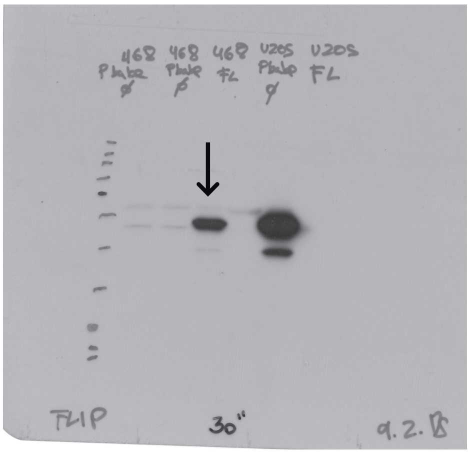

FLIP

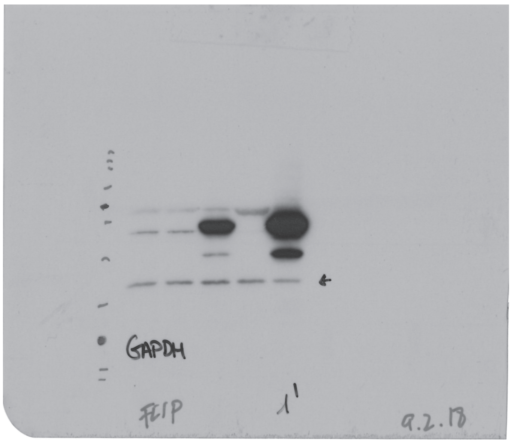

GAPDH

Figure 6D

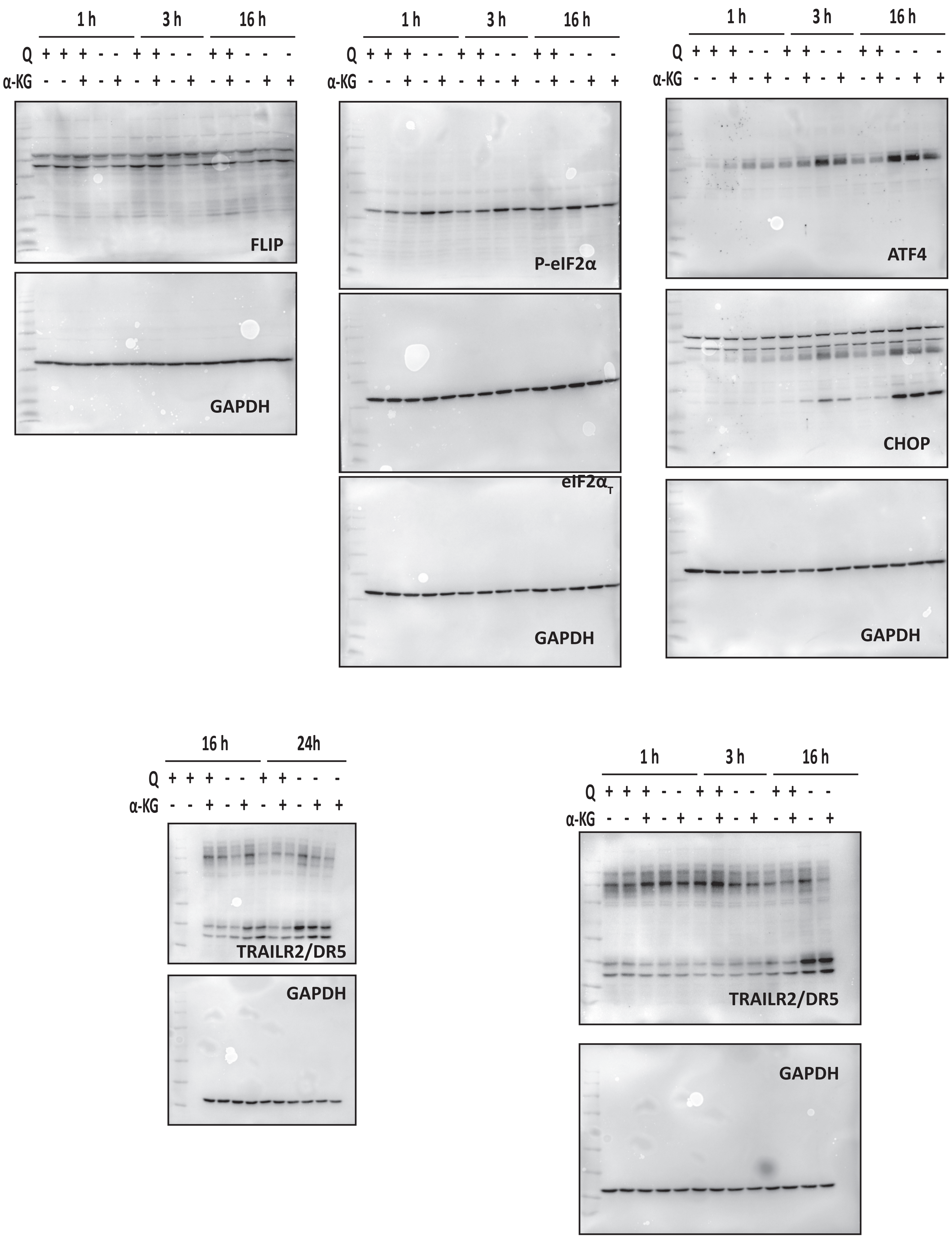

1 h
3 h
16 h
Q
α-KG
FLIP
GAPDH
P-eIF2α
eIF2α_T
GAPDH
ATF4
CHOP
GAPDH
16 h
24h
TRAILR2/DR5
GAPDH
TRAILR2/DR5
GAPDH

Figure 7B

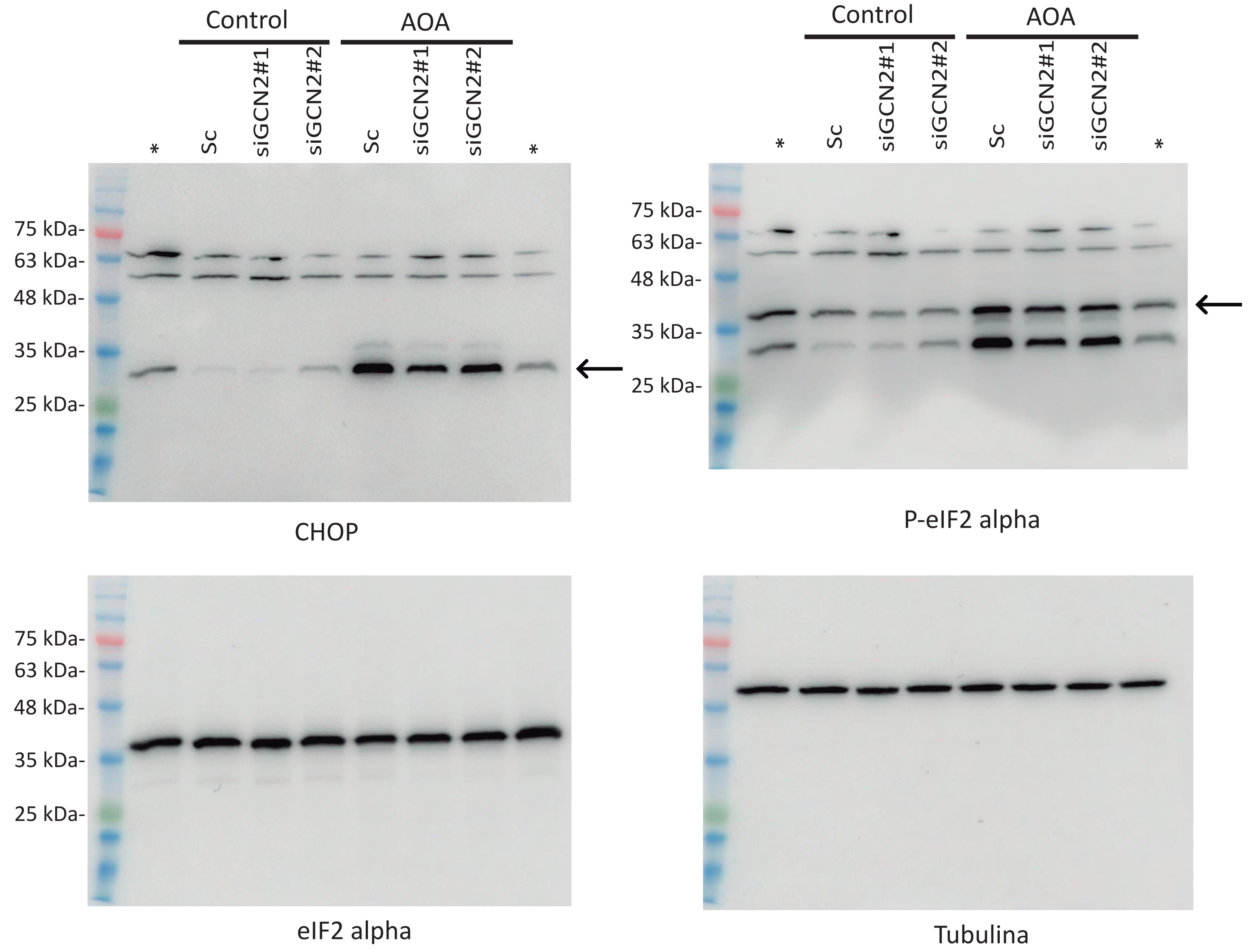

Figure 7D

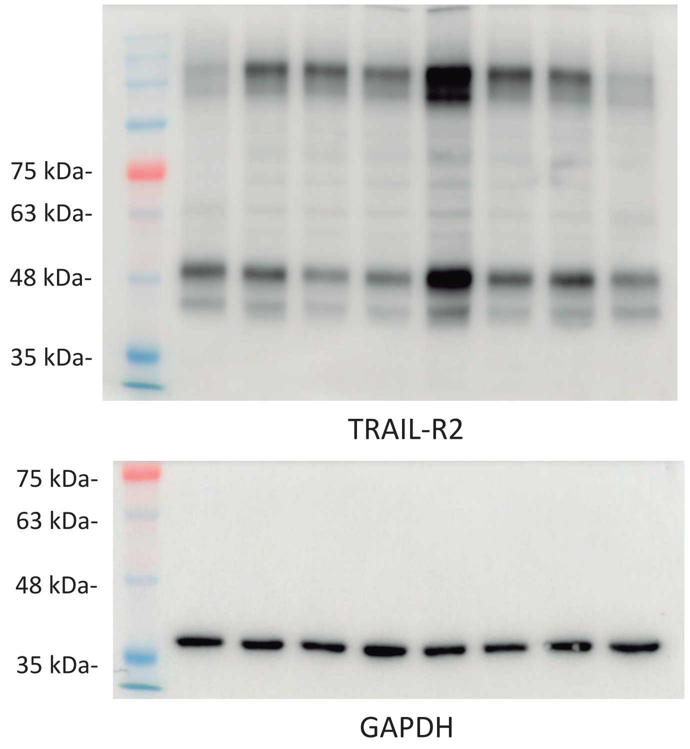

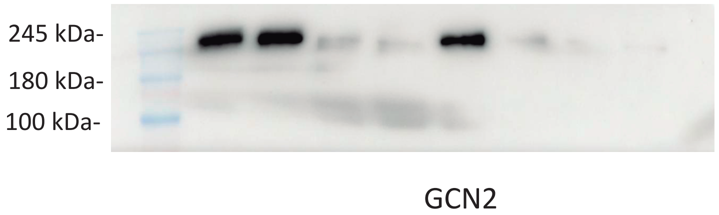

Figure 7E

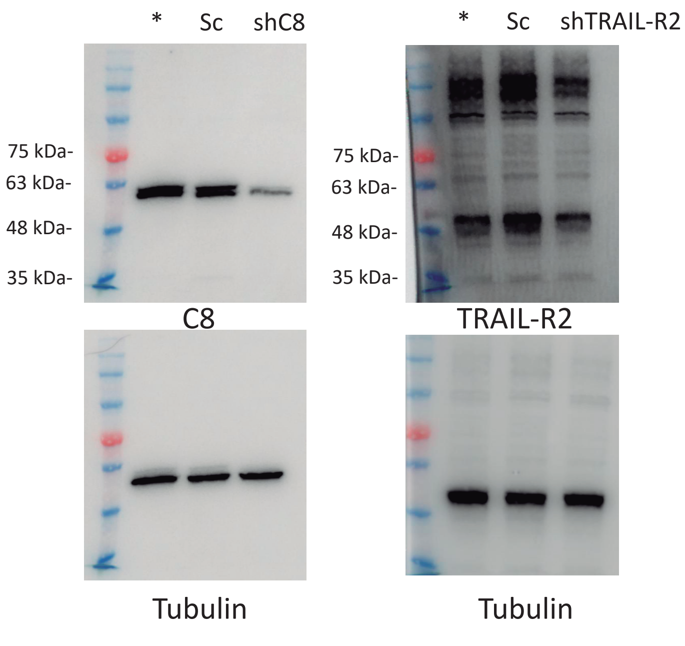

*Irrelevant sample

Figure S1 A

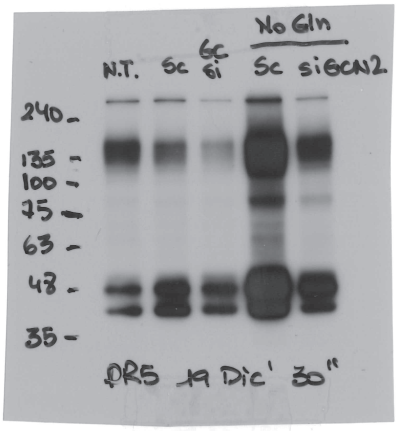

TRAIL-R2

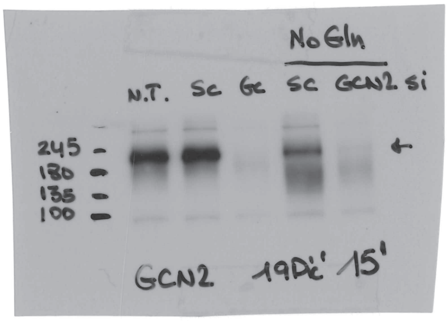

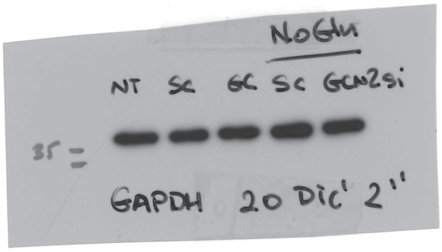

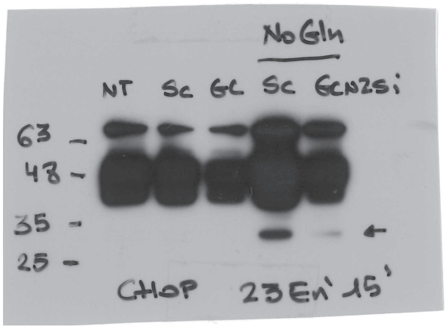

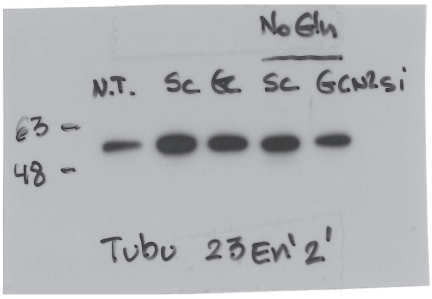

*Irrelevant sample

Figure S1 B

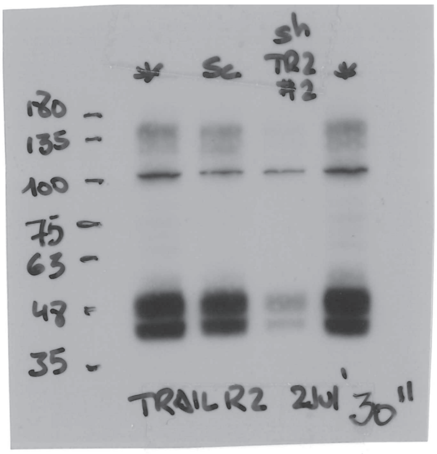

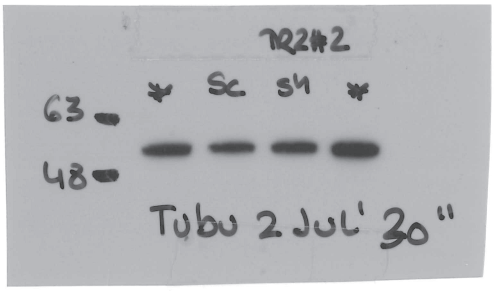

Figure S1 E

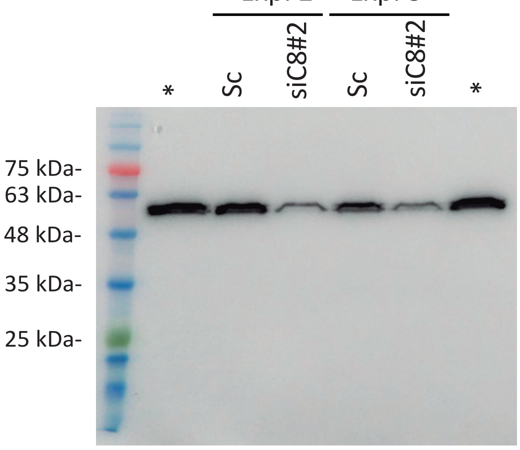

C8 XMS

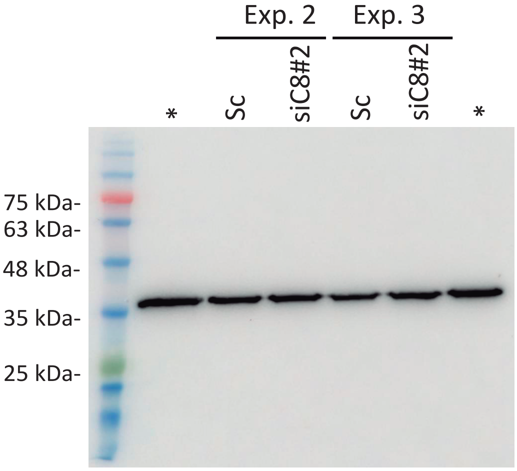

GAPDH

Figure S2A

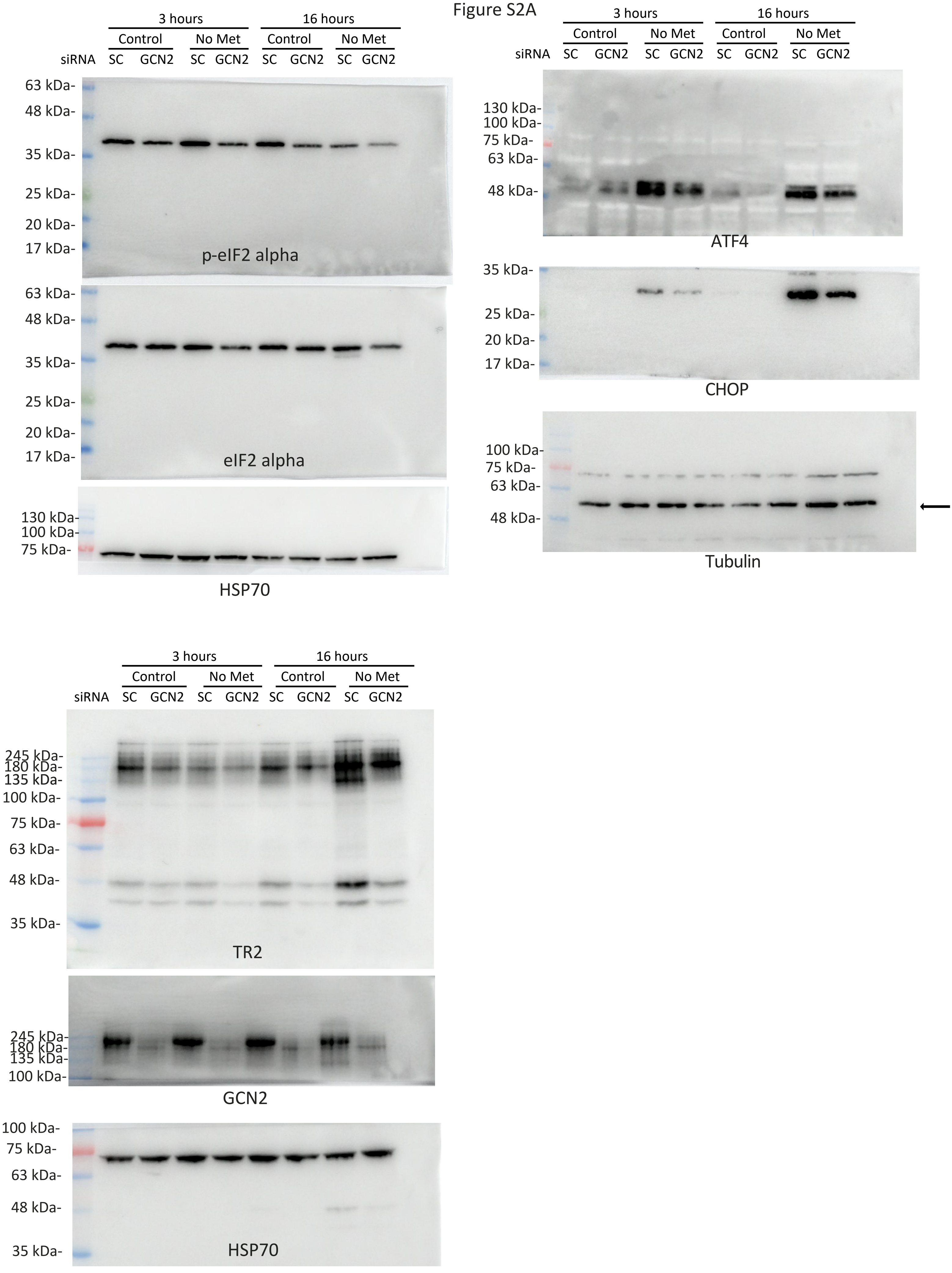
3 hours
16 hours
Control
No Met
siRNA
SC
GCN2
63 kDa-
48 kDa-
35 kDa-
25 kDa-
20 kDa-
17 kDa-
p-eIF2 alpha
eIF2 alpha
130 kDa-
100 kDa-
75 kDa-
HSP70
ATF4
CHOP
Tubulin
245 kDa-
180 kDa-
135 kDa-
TR2
GCN2

Figure S2B

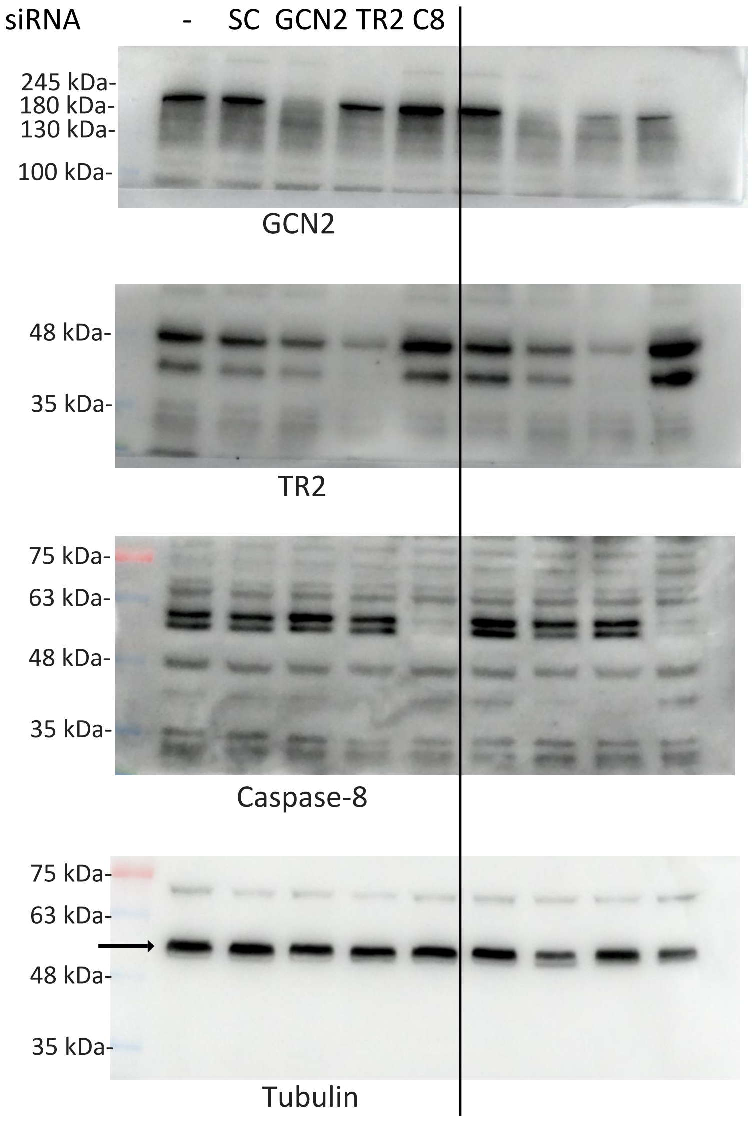
siRNA
-
SC
GCN2
TR2
C8
245 kDa-
180 kDa-
130 kDa-
100 kDa-
GCN2
48 kDa-
35 kDa-
TR2
75 kDa-
63 kDa-
48 kDa-
35 kDa-
Caspase-8
75 kDa-
63 kDa-
48 kDa-
35 kDa-
Tubulin

Figure S3A: left panel

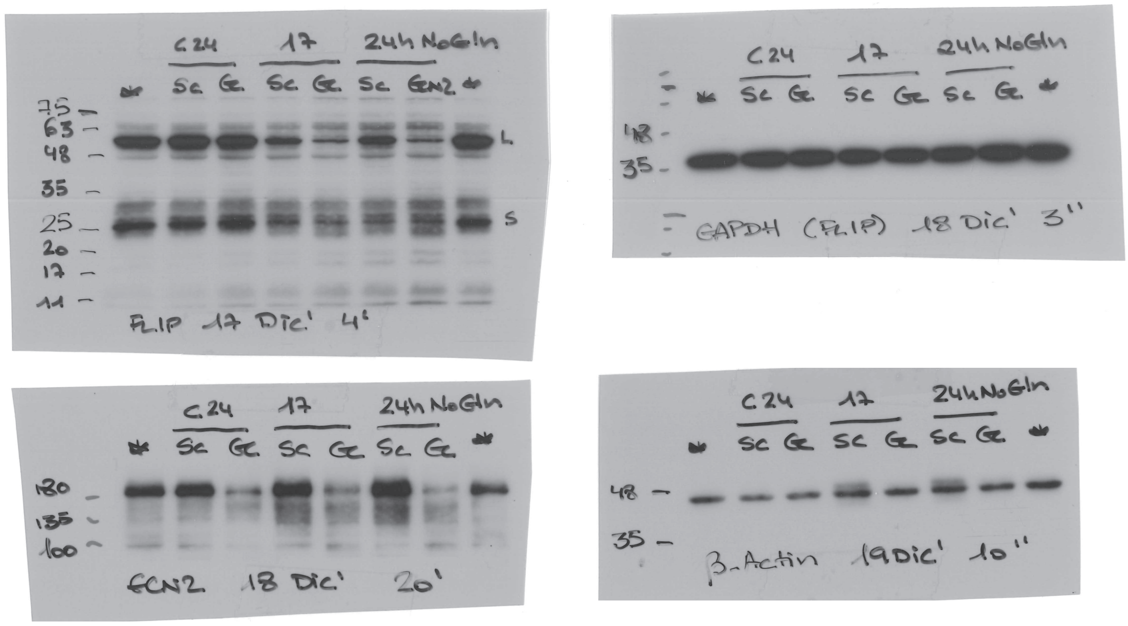

Figure S3A right panel

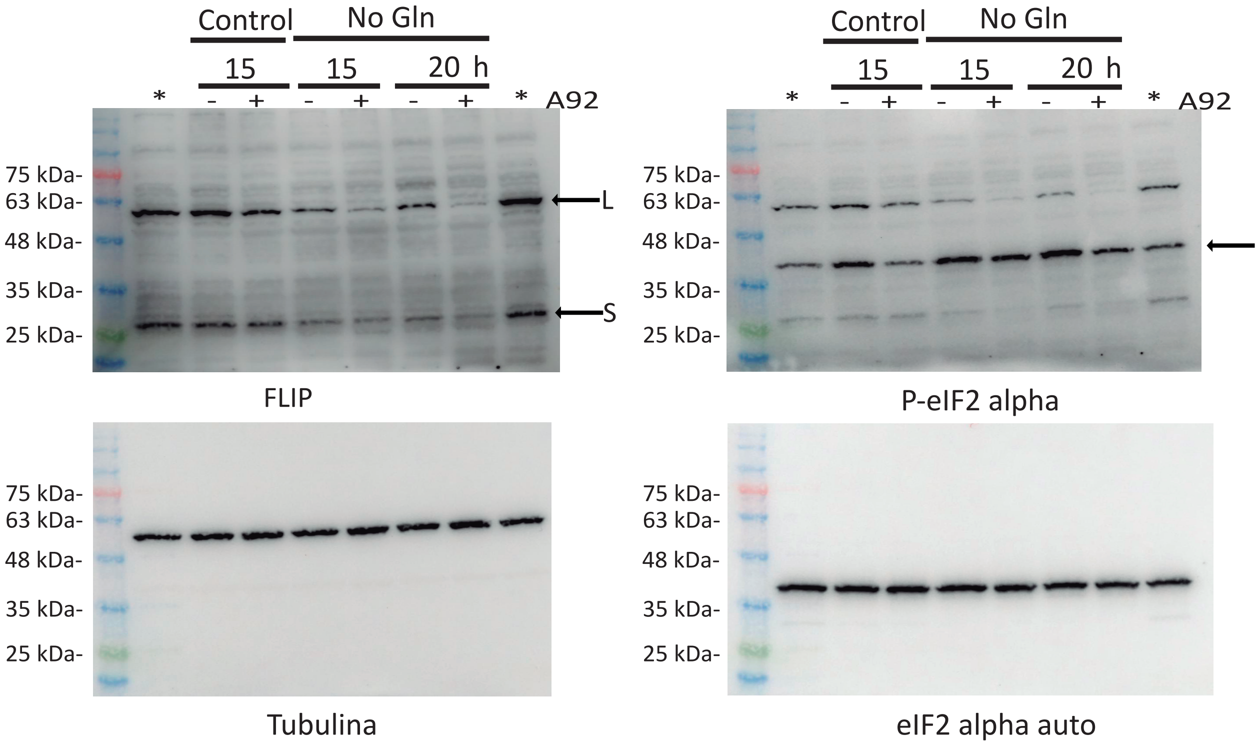

*Irrelevant sample

Figure S3B: upper panel

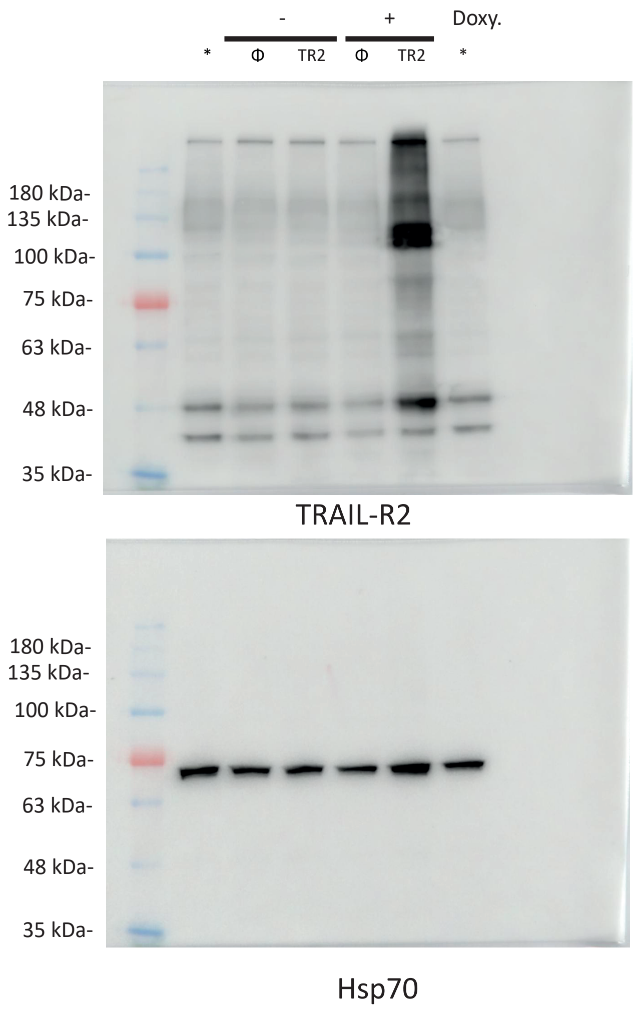

Figure S3B: lower panel

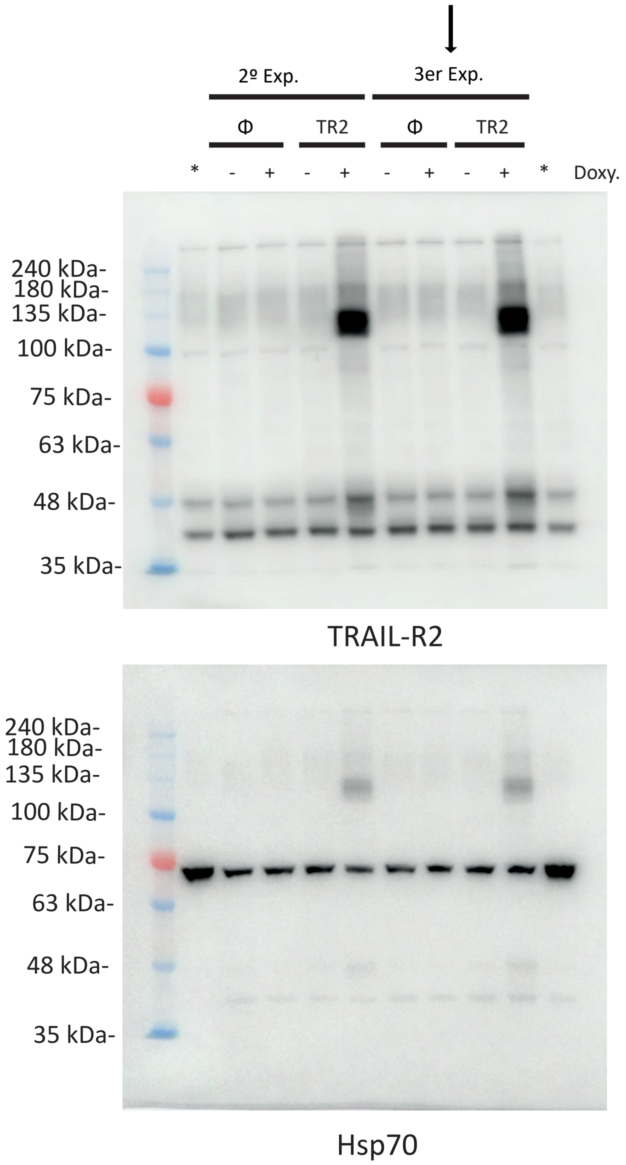

*Irrelevant sample

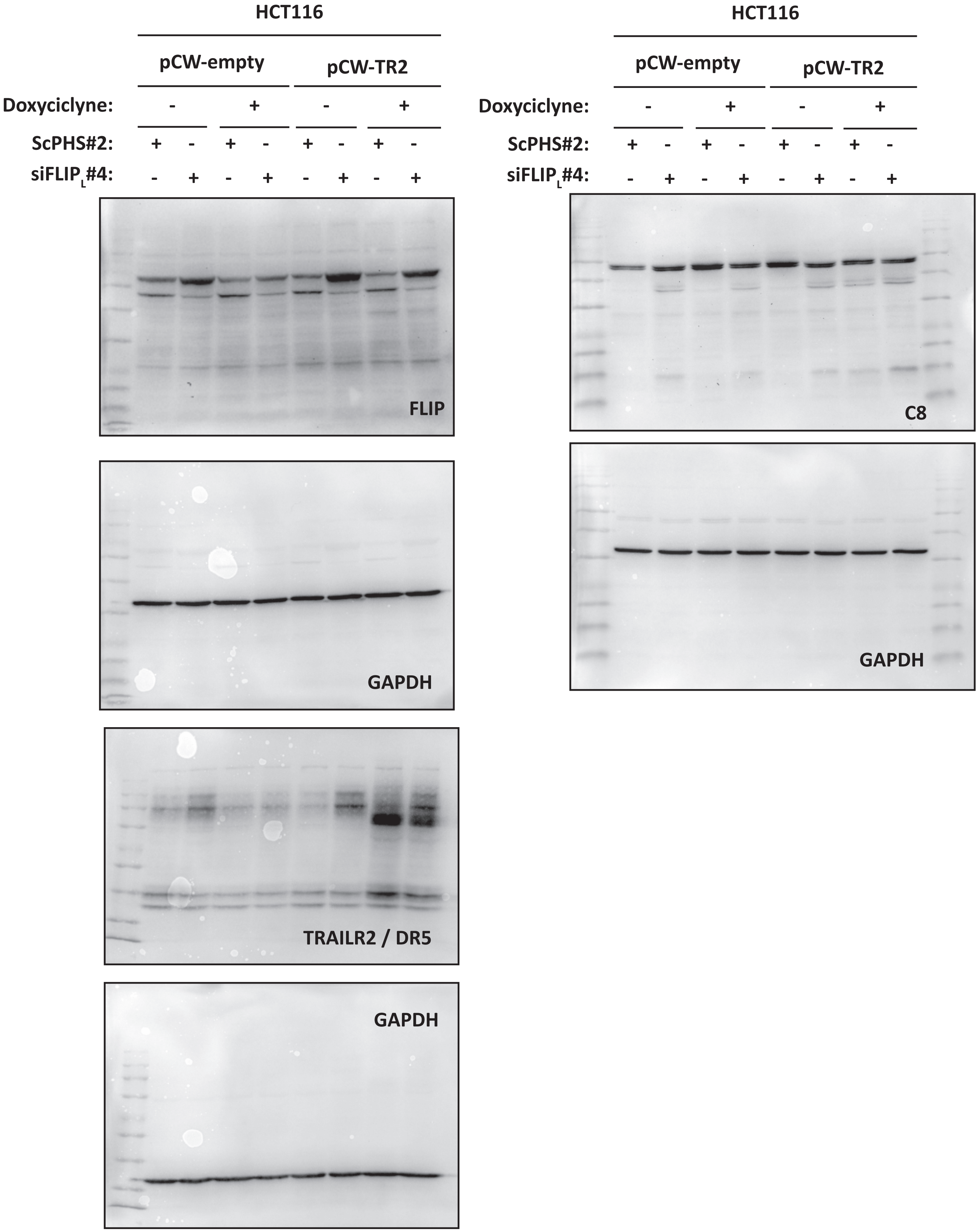

HCT116
pCW-empty
pCW-TR2
Doxyciclyne:
ScPHS#2:
siFLIP$_L$#4:
FLIP
GAPDH
TRAILR2 / DR5
GAPDH
HCT116
pCW-empty
pCW-TR2
Doxyciclyne:
ScPHS#2:
siFLIP$_L$#4:
C8
GAPDH

Figure S4A

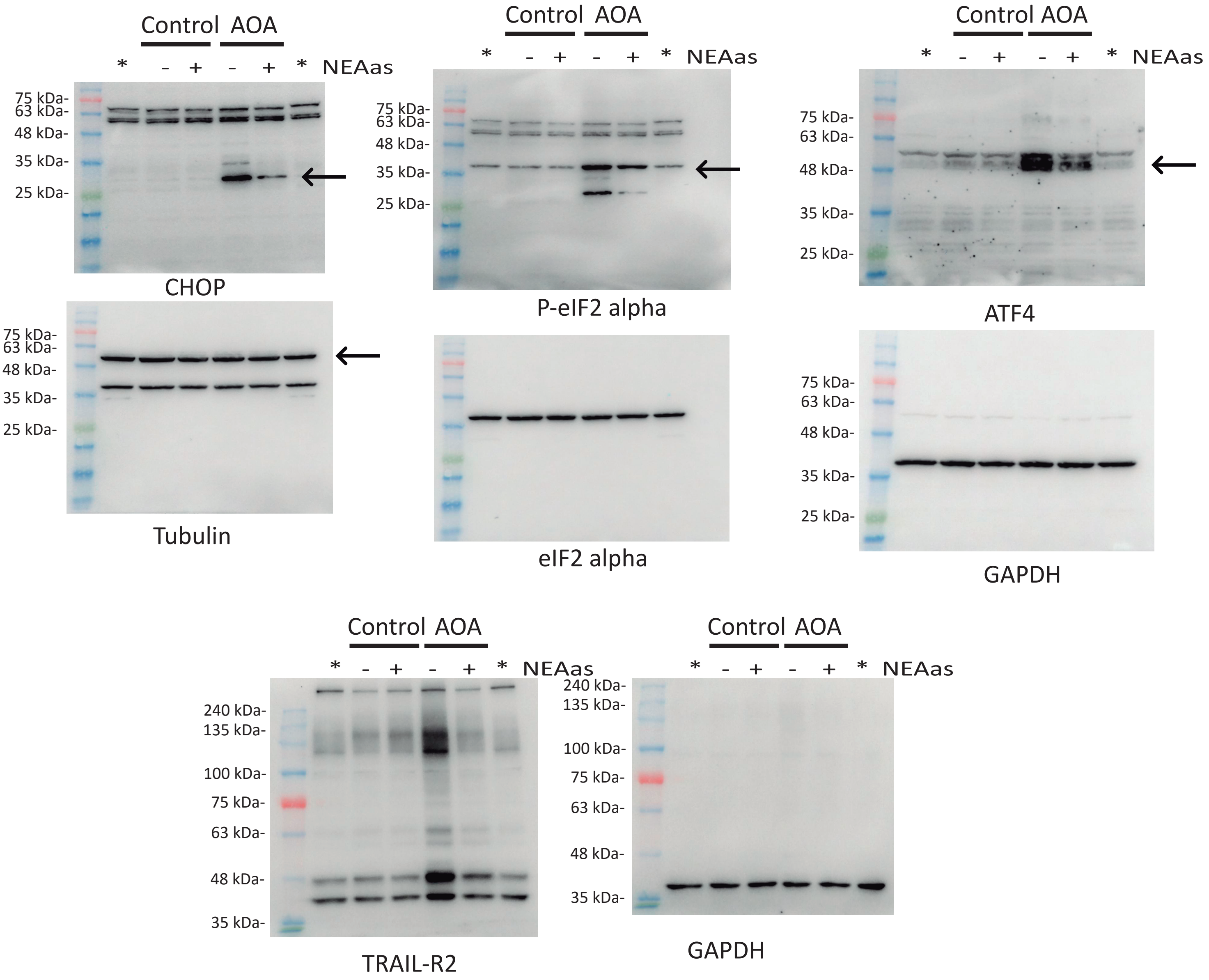

Figure S4B

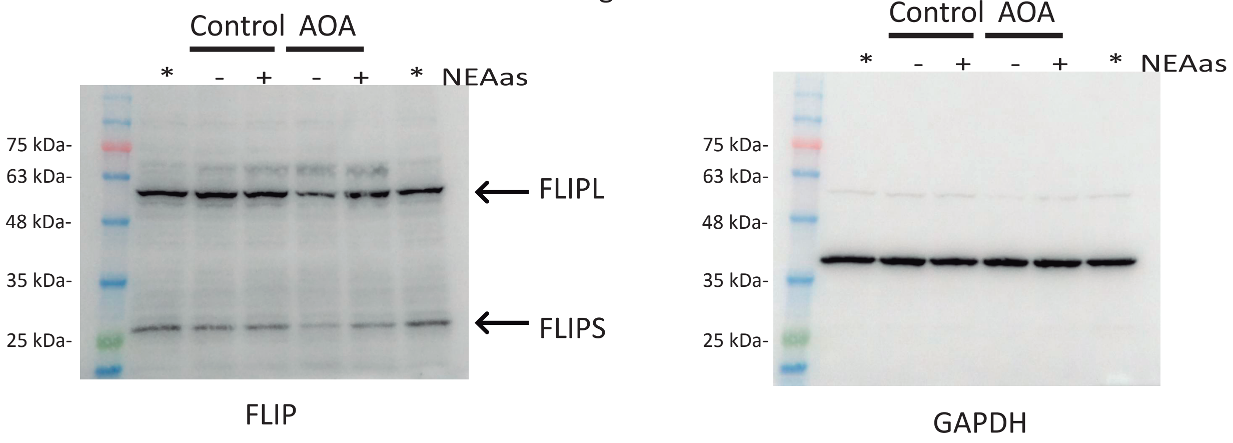

Figure S3C

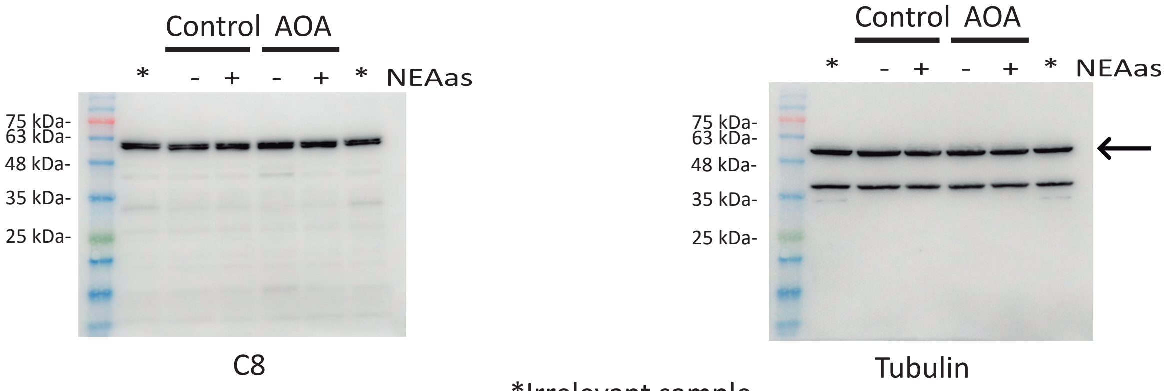

*Irrelevant sample
